# Expression of mitochondrial protein genes encoded by nuclear and mitochondrial genomes correlate with energy metabolism in dairy cattle

**DOI:** 10.1186/s12864-020-07018-7

**Published:** 2020-10-19

**Authors:** Jigme Dorji, Christy J. Vander Jagt, Josie B. Garner, Leah C. Marett, Brett A. Mason, Coralie M. Reich, Ruidong Xiang, Emily L. Clark, Benjamin G. Cocks, Amanda J. Chamberlain, Iona M. MacLeod, Hans D. Daetwyler

**Affiliations:** 1 School of Applied Systems Biology, La Trobe University, Bundoora, VIC 3083 Australia; 2 Agriculture Victoria, AgriBio, Centre for AgriBioscience, Bundoora, VIC 3083 Australia; 3 Agriculture Victoria, Ellinbank Dairy Centre, Ellinbank, VIC 3822 Australia; 4 Faculty of Veterinary & Agricultural Science, University of Melbourne, Parkville, VIC 3052 Australia; 5 The Roslin Institute and Royal (Dick) School of Veterinary Studies, University of Edinburgh, Edinburgh, Scotland UK

**Keywords:** Mitochondria, Energy metabolism, Differential gene expression, Gene co-expression, Cattle

## Abstract

**Background:**

Mutations in the mitochondrial genome have been implicated in mitochondrial disease, often characterized by impaired cellular energy metabolism. Cellular energy metabolism in mitochondria involves mitochondrial proteins (MP) from both the nuclear (*Nu*MP) and mitochondrial (*Mt*MP) genomes. The expression of MP genes in tissues may be tissue specific to meet varying specific energy demands across the tissues. Currently, the characteristics of MP gene expression in tissues of dairy cattle are not well understood. In this study, we profile the expression of MP genes in 29 adult and six foetal tissues in dairy cattle using RNA sequencing and gene expression analyses: particularly differential gene expression and co-expression network analyses.

**Results:**

MP genes were differentially expressed (DE; over-expressed or under-expressed) across tissues in cattle. All 29 tissues showed DE *Nu*MP genes in varying proportions of over-expression and under-expression. On the other hand, DE of *Mt*MP genes was observed in < 50% of tissues and notably *Mt*MP genes within a tissue was either all over-expressed or all under-expressed. A high proportion of *Nu*MP (up to 60%) and *Mt*MP (up to 100%) genes were over-expressed in tissues with expected high metabolic demand; heart, skeletal muscles and tongue, and under-expressed (up to 45% of *Nu*MP, 77% of *Mt*MP genes) in tissues with expected low metabolic rates; leukocytes, thymus, and lymph nodes. These tissues also invariably had the expression of all *Mt*MP genes in the direction of dominant *Nu*MP genes expression. The *Nu*MP and *Mt*MP genes were highly co-expressed across tissues and co-expression of genes in a cluster were non-random and functionally enriched for energy generation pathway. The differential gene expression and co-expression patterns were validated in independent cow and sheep datasets.

**Conclusions:**

The results of this study support the concept that there are biological interaction of MP genes from the mitochondrial and nuclear genomes given their over-expression in tissues with high energy demand and co-expression in tissues. This highlights the importance of considering MP genes from both genomes in future studies related to mitochondrial functions and traits related to energy metabolism.

## Background

There is growing evidence that mitochondrial dysfunction arises from variations in the mitochondrial genome and that their interplay with the nuclear genome has a role in mitochondrial diseases in humans, including metabolic disorders and diabetes [1–3]. Mitochondria and mitochondrial functions are critical for tissues with high energy requirement [4]. Energy is produced in mitochondria through a process of oxidative phosphorylation (OXPHOS). Besides energy production, mitochondria mediate programmed cell death (apoptosis), aging, calcium homeostasis, and signalling as reviewed in [5–7].

Mitochondrial proteins (MP) are the proteins localized in mitochondria and are key component to mitochondrial functions [8]. There are an estimated 1500 proteins in mitochondria of rats [9], participating as components of electron transport chain, metabolic pathways, and factors for replication, initiation and regulation in transcription and translation. To date, 1158 MP stand verified in human [10] and almost all MP (> 99%) are of nuclear origin (*Nu*MP) and imported into the mitochondria [11, 12] with the exception for 13 proteins (< 1%), which originate from the mitochondrial genome (*Mt*MP). Mitochondria have their own genome, which is inherited maternally [13–15]. The cattle mitochondrial genome is haploid with a small circular structure (~ 16.4 kb) with a coding region encoding for 37 genes (13 proteins, 22 tRNAs and 2 rRNAs) and a non-coding control region [16]. Mitochondrial DNA mutations in cattle have previously been shown to be associated with fertility and productivity [17–20], and environmental adaptability to high altitudes in yaks [21, 22]. Unlike nuclear DNA, mitochondrial genomes occur in multiple copies, and their numbers are relatively constant within a cell type and development stage but vary considerably among cell types [23–25].

Gene expression is referred to as one of a series of processes from gene activation to mature protein function that contributes to the expression of cellular phenotypes [26]. The expression of a gene is often specific to tissue types, and a notable example is the dominance of major milk protein transcripts in the bovine lactating mammary gland [27]. Gene expression is commonly studied using RNA sequencing (RNAseq) where the number of reads mapping to a gene (counts) is used to measure gene expression.

The characterization of gene expression, identification of gene function, gene-disease or gene-production associations from genome-wide gene expression [28] employs differential gene expression and co-expression network analyses. Differential gene expression compares the gene expression in a sample with another sample or group of samples. Gene co-expression analysis measures the correlation between the expression levels of genes and associates gene clusters with biological processes and facilitates prediction of gene function of previously unknown genes [29]. At a very local level, co-expression of small groups of genes results from being in close proximity [30, 31] and in chromosomal domains characterized by frequent internal DNA-DNA interactions known as topological association domains (TADs) [32].

Most RNAseq based gene expression analyses to date have focused on nuclear genes rather than genes from the mitochondrial genome [33]. Nonetheless, a comprehensive examination of MP genes from both genomes is central to understanding genome-genome interactions, their role in meeting specific energy demand, and development of mitochondrial diseases. Metabolic profiles and energy demands vary widely across organs and tissue types [34–36]. The varying demand for energy across tissues is possibly in part facilitated through tissue specific and differential expression of MP genes. Currently, a comprehensive study on the expression of MP genes (both *Nu*MP and *Mt*MP) across tissues is lacking in bovine, although the expression of individual or groups of *Mt*MP genes has been published as part of larger gene sets [33, 37]. Therefore, our study aimed to characterize MP gene expression across both adult and foetal tissues in dairy cattle. We used RNAseq of 35 tissues from two adult cows and two foetuses (29 adult and six foetal tissues) to investigate differential gene expression and gene co-expression. We validated our findings using publicly available RNAseq data for an additional dairy cow and three sheep.

## Results

### Differential expression of mitochondrial protein genes

#### Main cows: adult

**Fig. 1:**
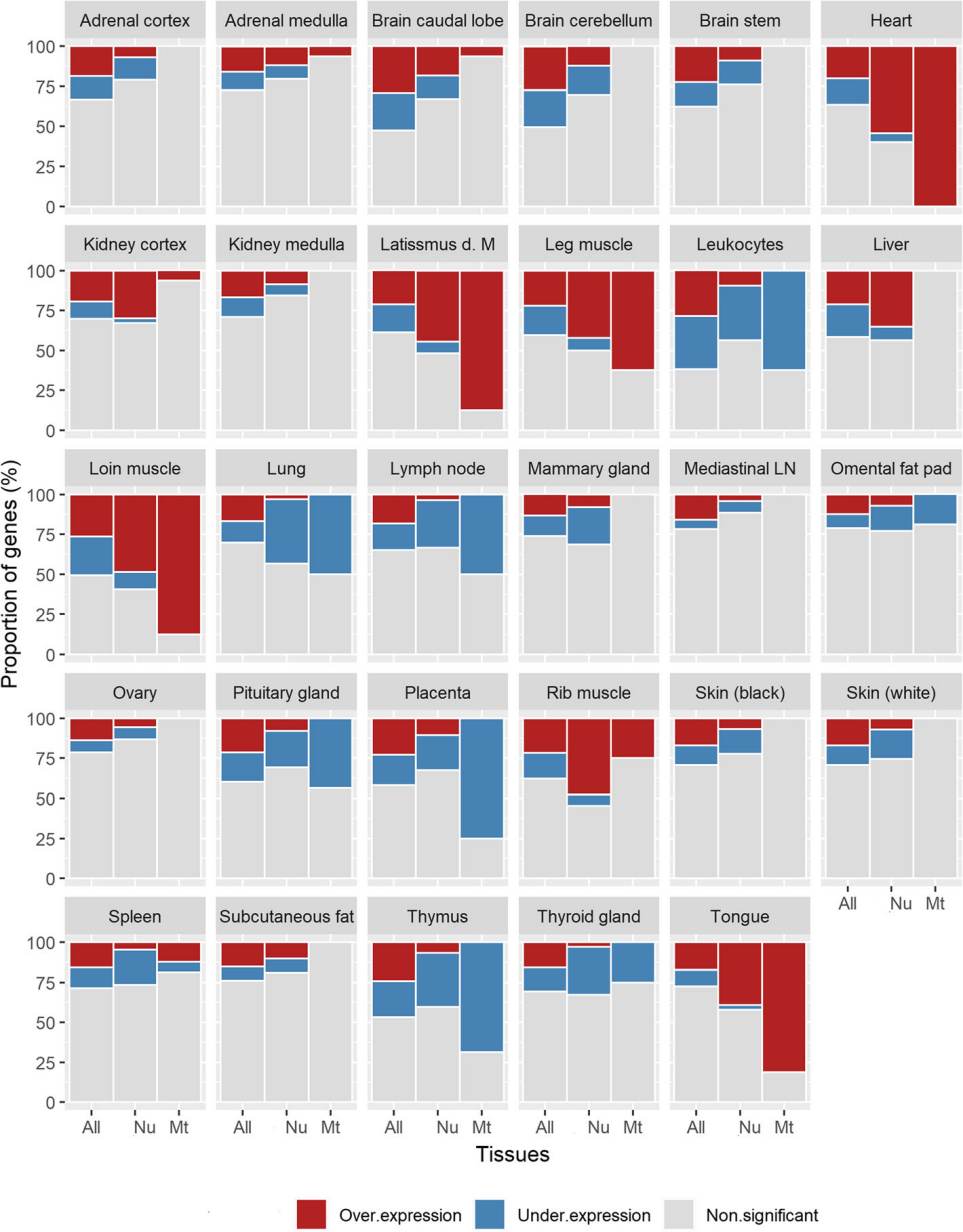
Percentage of differentially expressed genes by gene categories for 29 tissues in the Main Cows dataset. m. = muscle, LN = lymph node; Gene category: All = All protein coding genes from nuclear and mitochondrial genomes, Nu = Mitochondrial protein coding genes from the nuclear genome (*Nu*MP), Mt = Mitochondrial protein coding genes from the mitochondrial genome (*Mt*MP)

In total, 16,166 genes including 1041 MP genes were available for analysis after filtering (out of 24,616 annotated Ensembl genes). A gene was considered as differentially expressed (DE) in a tissue if the expression was different from the average expression across all other tissues (LFC > |0.6|, FDR < 0.01). Across all genes, 13 to 40% of genes in total were DE in one or more tissues and as high as 50% each of the DE genes were over-expressed or under-expressed (Fig. 1). Table 1 provides a summary of the number of DE genes by category across tissues. The highest overall numbers of DE genes among the tissues were in blood leukocytes(*N* = 9218), loin muscle (*N* = 7560), brain caudal lobe (*N* = 7504), and brain cerebellum (*N* = 7161), and the lowest in the ovary (*N* = 3003), omental fad pad (*N* = 3008), and mediastinal lymph node (*N* = 3428). The DE genes in heart, skeletal muscles and tongue were significantly enriched for OXPHOS, metabolic pathways and neurodegenerative diseases pathways, and enriched for metabolic pathways in liver and kidney cortex (Table 2).

**Table 1:** Number of differentially expressed (DE) genes in tissues by gene categories averaged for two cows in the Main Cows dataset

Tissue	MG (37)			***Nu***MP (1041)			All (24,616)			***Mt***MP (13)	***Mt*** tRNA	***Mt*** rRNA
	Over	Under	Total DE	Over	Under	Total DE	Over	Under	Total DE			
Adrenal cortex	0	0	0	66	157	223	2018	2532	4550	0	0	0
Adrenal medulla	2	0	2	124	89	213	1620	2260	3880	0	0	2
Brain caudal lobe	2	0	2	190	151	341	3354	4150	7504	0	0	2
Brain cerebellum	1	0	1	120	187	307	3282	3879	7161	0	0	1
Brain stem	0	0	0	95	161	256	1911	3294	5205	0	0	0
Heart	17	0	17	576	53	629	2185	2833	5018	13	2	2
Kidney cortex	5	0	5	315	30	345	1465	2851	4316	3	0	2
Kidney medulla	0	0	0	93	58	151	1618	2444	4062	0	0	0
Latissimus dorsi M.	14	0	14	481	74	555	2668	2970	5638	13	1	0
Leg muscle	14	0	14	456	80	536	2773	3083	5856	13	1	0
Leukocytes	0	9	9	99	368	467	5270	3948	9218	9	0	0
Liver	0	0	0	360	82	442	2914	3124	6038	0	0	0
Loin muscle	14	0	14	514	114	628	3853	3707	7560	13	1	0
Lung	0	5	5	31	430	461	1793	2565	4358	5	0	0
Lymph node	0	7	7	38	306	344	2649	2562	5211	7	0	0
Mammary	0	0	0	86	250	336	1805	1864	3669	0	0	0
Mediastinal LN	0	0	0	46	77	123	993	2432	3425	0	0	0
Omental fat	0	2	2	78	163	241	1107	1901	3008	0	0	2
Ovary	0	0	0	58	70	128	985	2018	3003	0	0	0
Pituitary gland	0	5	5	73	251	324	2522	3033	5555	5	0	0
Placenta	0	10	10	110	232	342	2842	3267	6109	10	0	0
Rib muscle	7	0	7	500	70	570	2325	2933	5258	7	0	0
Skin black	0	0	0	79	176	255	1814	2574	4388	0	0	0
Skin white	0	0	0	79	205	284	1780	2554	4334	0	0	0
Spleen	2	0	2	47	260	307	2003	2449	4452	0	0	2
Subcutaneous fat	0	0	0	105	95	200	1299	2238	3537	0	0	0
Thymus	0	10	10	63	363	426	3509	3208	6717	10	0	0
Thyroid	0	2	2	33	329	362	2335	2335	4670	2	0	0
Tongue	14	0	14	413	30	443	1369	2365	3734	13	1	0

() total number of genes in a category; Over = Over-expression, Under = under-expression; MG  Genes from mitochondrial genome including tRNA and rRNAs, *Nu*MP  Mitochondrial protein genes encoded by the nuclear genome, *Mt*MP  Mitochondrial protein genes encoded by the mitochondrial genome, *Mt* tRNA  Mitochondrial transfer RNA, *Mt* rRNA  Mitochondrial ribosomal RNA, All  all genes from nuclear and mitochondrial genomes, *M* Muscle, *LN* Lymph node

**Table 2:** KEGG functional annotation of overall differentially expressed genes of selected tissues with the largest number of genes averaged across two cows in the Main Cows dataset

Tissues	Enrichment	No. of genes (Overlap ***Nu***MP^**a**^)	Adj. p	Other pathways
Heart	Oxidative phosphorylation	101 (93)	3.3e^−53^	Parkinson’s disease, Alzheimer’s disease, Huntington’s disease, NAFLD, carbon metabolism, Metabolic pathways, cardiac muscle contraction, TCA cycle,
Leg muscle	Oxidative phosphorylation	83 (38)	1.0e^−32^	Parkinson’s disease, Alzheimer’s disease, NAFLD, Huntington’s disease, Carbon metabolism, metabolic pathways, Proteasome, cardiac muscle contractionBiosynthesis of antibiotics
Loin muscle	Oxidative phosphorylation	89 (86)	5.8e^− 39^	Parkinson’s disease, Alzheimer’s disease, NAFLD, Huntington’s disease, Huntington’s disease, carbon metabolism, Proteosome, Cardiac muscle contraction
Rib muscle	Oxidative phosphorylation	86 (39)	1.8e^−35^	Parkinson’s disease, Alzheimer’s disease, NAFLD, Huntington’s disease, Metabolic pathways, carbon metabolism, Proteasome
Tongue	Oxidative phosphorylation	98 (91)	3.0e^−50^	Parkinson’s disease, Alzheimer’s disease, NAFLD, Huntington’s disease, Metabolic pathways, carbon metabolism, cardiac muscle contraction,
Kidney cortex	Metabolic pathways	312 (118)	3.9e^−28^	Biosynthesis of antibiotics, Carbon metabolism, Valine, leucine and isoleucine degradation, Glycine, serine and threonine metabolism, tryptophane metabolism, Fatty acid metabolism
Kidney medulla	Focal adhesion	67 (0)	3.0e^−10^	Tight junction, calcium signalling pathway, Gastric acid secretion. ECM-receptor interaction, cGMP-PKG signalling pathway, Gastric acid secretion, Dilated cardiomyopathy
Liver	Metabolic pathways	387 (123)	4.1e^−49^	Biosynthesis of antibiotics, Peroxisome, valine, leucine and isoleucine degradation, complement and coagulation cascades, fatty acid degradation, tryptophan metabolism, carbon metabolism
Brain caudal lobe	Axon guidance	57 (0)	8.0e^−18^	Glutamatergic Synapse, Domaminergic synapse, MAPK signalling pathway, Adrenergic signalling in cardiomyocytes, Retrograde endocannabinoid signalling, cAMP signalling pathway, Synaptic vesicle cycle, GABAergic synapse, Morphine addiction, Glutamatergic synapse, Circadian entrainment, Dopaminergic synapse
Brain cerebellum	Glutamatergic synapse	49 (2)	3.1e^− 14^	GABAergic synapse, Retrograde endocannabinoid signalling, Morphine addiction, Circadian entrainment, Dopaminergic synapse, cAMP signalling pathway, Adrenergic signalling in cardiomyocytes, axon guidance,
Brain stem	GABAergic synapse	36 (2)	3.1e^− 9^	Glutamergic synapse, Morphine addiction, Retrograde endocannabinoid signalling, Dopaminergic synapse, Circadian entrainment
Leukocytes	Chemokine signalling pathways	74	4.7e^−15^	Focal adhesion, leukocyte transendothelial migration, Rap1 signalling pathway, natural killer cell mediated cytotoxicity, regulation of actin cytoskeleton, B cell receptor signalling pathway

^**a**^we show the number of genes in the top enriched pathway that overlap mitochondrial proteins

**Fig. 2:**
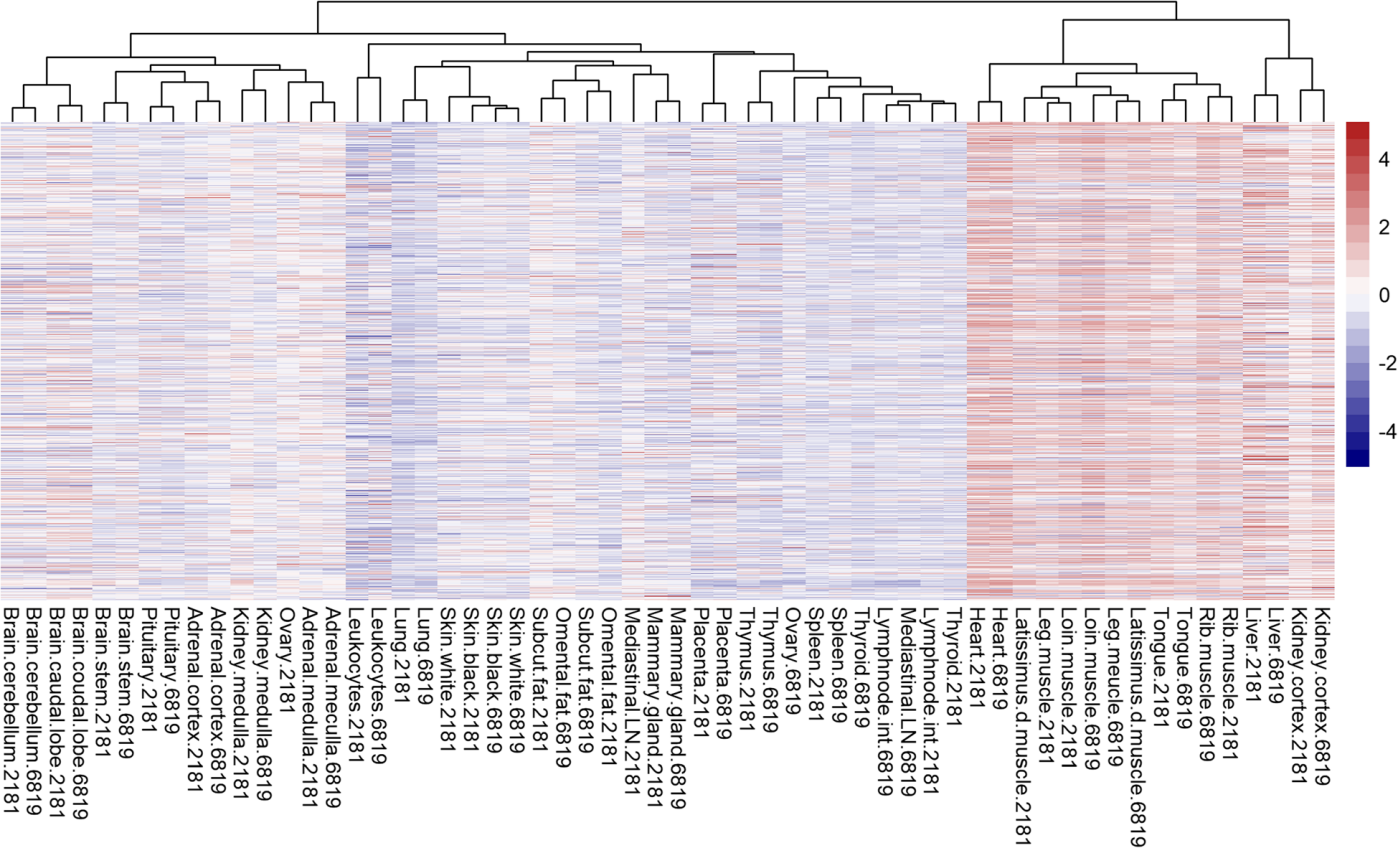
Heatmap of nuclear genome encoded mitochondrial protein (*Nu*MP) gene expression the Main Cows dataset. 6819 and 2181 are Cow No. 6819 and Cow No. 2181 respectively

More than 99% of the MP genes originate from the nuclear genome (*Nu*MP). The proportion of DE *Nu*MP genes across the tissues varied from 12 to 60% with higher proportions (> 50%) in heart and skeletal muscles. The proportion of under or over-expressed DE *Nu*MP genes within tissues varied considerably. A relatively greater proportion of *Nu*MP genes were over-expressed in heart, kidney cortex, skeletal muscles, and tongue, and under-expressed in blood leukocytes, lymph nodes, placenta, lungs, mammary, and thymus (Fig. 2). The expression of *Nu*MP genes was similar between animals in the Main Cows as indicated by the clustering together of same tissues, with the exception of five tissues (Fig. 2; adipose, ovary, kidney cortex, and leukocytes).

**Fig. 3:**
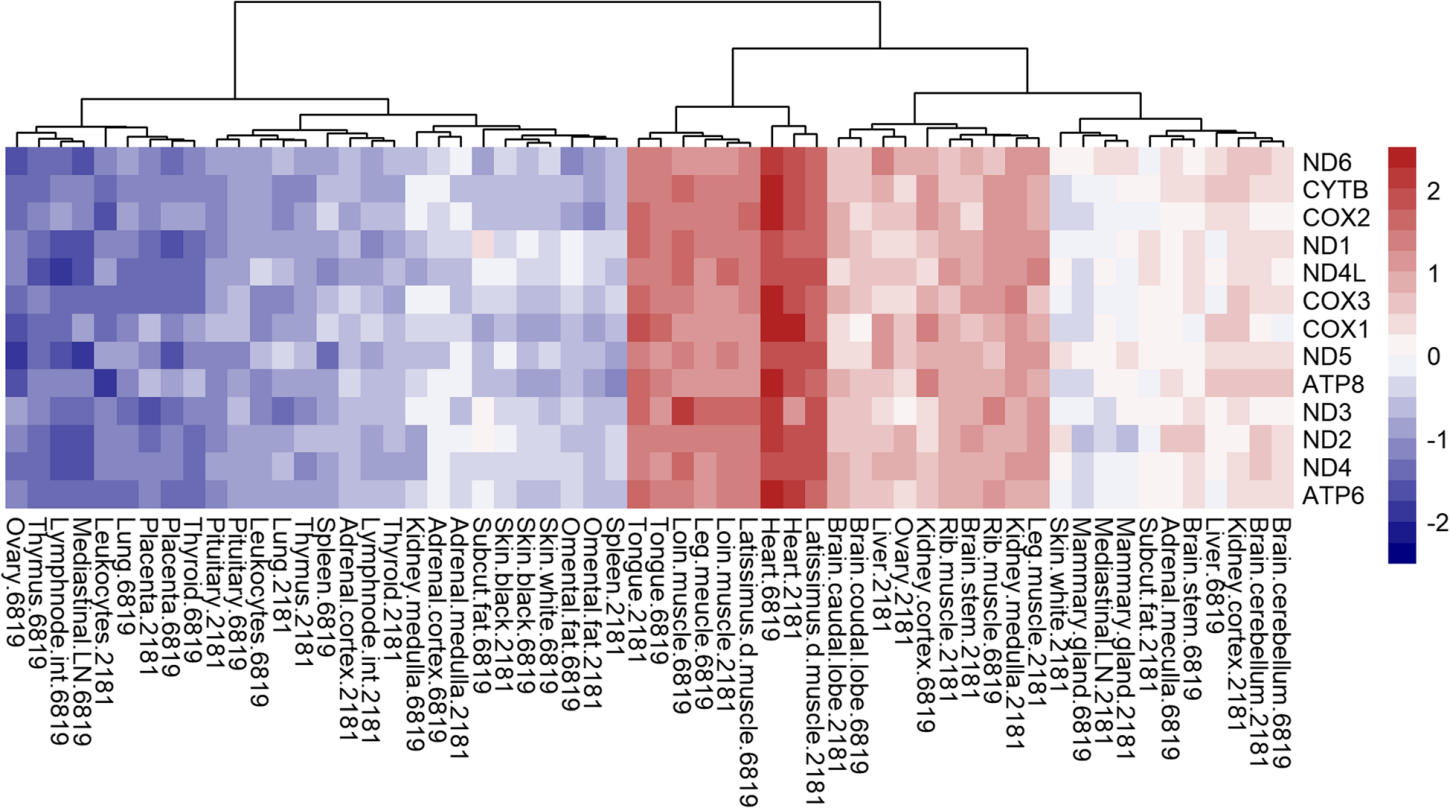
Heatmap of mitochondrial genome encoded mitochondrial protein (*Mt*MP) gene expression in the Main Cows dataset. Based on the log_2_ counts per million of *Mt*MP genes across 29 tissues from cows 6819 and 2181

In contrast to *Nu*MP, differential expression of *Mt*MP genes were observed in less than 50% of tissues (14 out of 29 tissues). The proportion of DE *Mt*MP genes within tissues ranged widely from 0 (no genes) to 100% (all 13 *Mt*MP genes). Specifically, *Mt*MP genes were 100% DE in heart, leg muscle, latissimus dorsi muscle, loin muscle, and tongue, and ranged between 50 and 75% in other tissues (leukocytes, placenta, thymus, rib muscle, and lymph node). Unlike *Nu*MP genes, all DE *Mt*MP genes were expressed in a single direction (i.e. either all over-expressed or all under-expressed) meaning every DE *Mt*MP gene was over-expressed in heart, tongue, muscles and kidney cortex, and under-expressed in blood leukocytes, placenta, lymph node, pituitary, thymus, and thyroid (Fig. 3). Further, there were similarities between the expression of DE *Mt*MP and *Nu*MP genes within a tissue. For instance, every tissue showing over-expression of DE *Mt*MP genes invariably showed predominant over-expression of *Nu*MP genes and similarly for under-expression. In addition to *Mt*MP genes, some of the non-protein coding genes from the mitochondrial genome were also DE in several tissues (*16 s rRNA*, *12 s rRNA*, *tRNA-Pro* and *tRNA-Ser)*.

Within groups of tissues with either over-expression of MP genes (heart, skeletal muscles, liver and kidney cortex) or under-expression (leukocytes, thymus, placenta and lymph node), we examined all overlapping genes and their functional enrichment. In heart and skeletal muscles, there were 1088 over-expressed genes in common including 320 *Nu*MP and seven *Mt*MP genes. Altogether across these 1088 genes, there was significant enrichment for OXPHOS, metabolic pathways and neurodegenerative disease pathways as in these individual tissues. Similarly, liver and kidney cortex had 1249 over-expressed genes in common including 223 *Nu*MP genes (0 *Mt*MP genes) and these were significantly enriched for metabolic pathways and peroxisome, valine, leucine and isoleucine degradation. In contrast, the DE genes in common for tissues in the under-expression group was low (63 genes) with only 20 *Nu*MP genes (0 *Mt*MP genes). Across all 63 genes, there was enrichment for adrenergic signalling in cardiomyocytes, dilated cardiomyopathy, cardiac muscle contraction and hypertrophic cardiomyopathy. Altogether, these results indicated a significant role of the over-expressed MP genes contributing to the enriched pathways in the over-expression tissue group, while this pattern was not observed in the under-expression group.

#### Main cows: Foetuses

The analysis for functional enrichment of overall DE genes in six foetal tissues showed significant enrichment of OXPHOS and metabolic pathways only in heart and lungs but not in leg muscle (Additional file 6). The *Nu*MP genes were over-expressed in the heart and under-expressed in the remaining tissues, including leg muscles (Additional file 7). Similarly, the *Mt*MP genes were prominently over-expressed in heart, under-expressed in the lungs and not significant in the remaining tissues (Additional file 8). Higher expression of *Nu*MP genes was observed in liver of the male foetus and it did not cluster with liver of the female foetus.

### Co-expression network analysis of mitochondrial protein genes

**Fig. 4:**
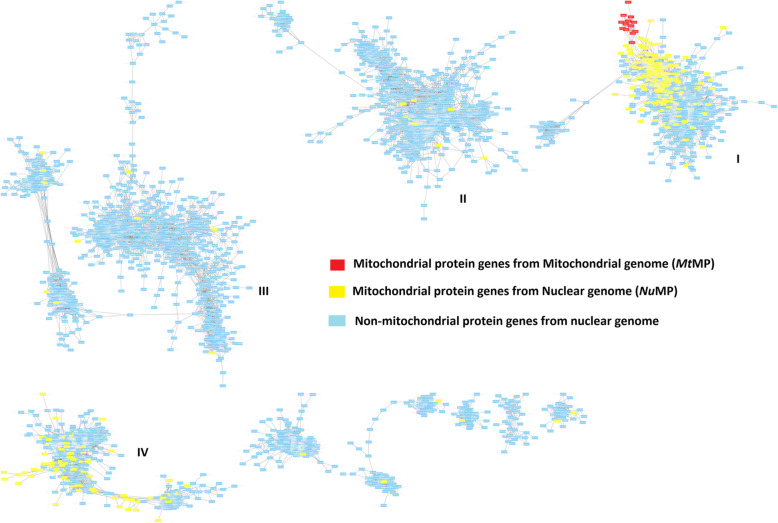
Gene co-expression network clusters across tissues in the Main Cows based on similarity matrix computed using Pearson correlations > |0.95|

The gene co-expression network constructed based on the affinity matrix from genes correlated in expression > |0.95| in adult cows had altogether 3643 genes clustered into four major network clusters I-IV (Fig. 4). The *Nu*MP genes were concentrated in two main clusters (I and IV) indicating co-expression among *Nu*MP genes and the remaining *Nu*MP genes were sparsely scattered across all other clusters. Similarly, *Mt*MP genes were all grouped in cluster I. Clusters I and IV containing subgroups of highly co-expressed *Nu*MP and *Mt*MP and *Nu*MP genes are referred to as *Nu*MP-*Mt*MP and *Nu*MP clusters respectively. Within the *Nu*MP-*Mt*MP cluster, the MP genes from the respective genomes were highly co-expressed. The *Nu*MP-*Mt*MP cluster was significantly enriched for OXPHOS, metabolic pathways and mitochondrial diseases’ pathways. Similarly, the *Nu*MP cluster (cluster IV) was over-represented for signalling pathways, contraction, metabolic pathways and myopathies related to the heart (Table 3). The gene functions of the non-mitochondrial protein genes (Non-MP) in the *Nu*MP-*Mt*MP cluster were associated with heart and muscle functioning, signalling, and contraction (Additional files 9,10).

**Table 3:** Summary of gene, composition, and functional enrichment of KEGG pathways of genes in co-expression clusters (FDR <1e-05) in the Main Cows dataset

Cluster	No. of ***Mt***MP genes	No. of ***Nu***MP Genes	No. of Non-MP genes	Total No. of genes	Enrichment of pathways
I (*Nu*MP-*Mt*MP Cluster)	13	216	584	813	Parkinson’s disease, Oxidative phosphorylation, Alzheimer’s diseases, Huntington diseases, Non-alcohol fatty liver diseases, metabolic pathways, Citrate cycle, carbon metabolism, Cardiac muscle contraction, Proteosome
II	0	10	871	881	Retrograde endocannabinoid signaling, GABAergic synapse, Nicotine addiction, Morphine addictions, Glutamatergic synapse, Dopaminergic synapse, Synaptic vesicle cycle, Neuroactive ligand-receptor interaction
III	0	12	923	935	Cell adhesion molecules (CAMs), *Staphylococcus aureus* infection, intestinal immune network for IgA production, Leishmaniasis, Antigen processing and presentation, viral myocarditis, Allograft rejection, primary immunodeficiency, Hematopioetic cell lineage, Natural killer cell-mediated cytotoxicity
IV (*Nu*MP cluster)	0	79	466	545	Chemical carcinogenesis, Complement and coagulation cascade, Drug metabolism – cytochrome p450 metabolism, steroid hormone biosynthesis, retinol metabolism, Metabolic pathways, Complement and coagulation cascades, bile secretion, primary bile acid biosynthesis, tryptophan metabolism, carbon metabolism, fatty acid metabolism

We tested if the co-expression of *Nu*MP genes in the *Nu*MP-*Mt*MP cluster was due to random chance using a Chi-square goodness of fit test. The frequency of *Nu*MP genes in the cluster was significantly higher than random (^2^ = 307.6, *p* < 0.01), supporting that the cluster was enriched with co-expressed MP.

Further, we investigated the effect of TAD on the co-expression by comparing the number of 651 TAD mapped genes in the *Nu*MP-*Mt*MP cluster with the mean from 100 randomly generated samples of 651 genes from 3022 TAD mapping genes across the clusters. It showed involvement of *Nu*MP-*Mt*MP genes in a similar number of TADs (472 ± 10 vs 484), but *Nu*MP-*Mt*MP were more likely to be present in groups of two or more within TADs. The total number of genes occurring in a two or more in a TAD was 282 and 116 (±6) in *Nu*MP-*Mt*MP cluster and random samples respectively. This indicated that co-expression in the *Nu*MP-*Mt*MP cluster was enriched within TADs.

### Validation of patterns of mitochondrial protein gene expression

The key findings on the expression of MP genes from the Main Cows dataset were validated using two independent datasets (Validation Cow and Validation Sheep). Both validation sets confirmed the general trends of MP gene expression and co-expression in tissues.

**Fig. 5:**
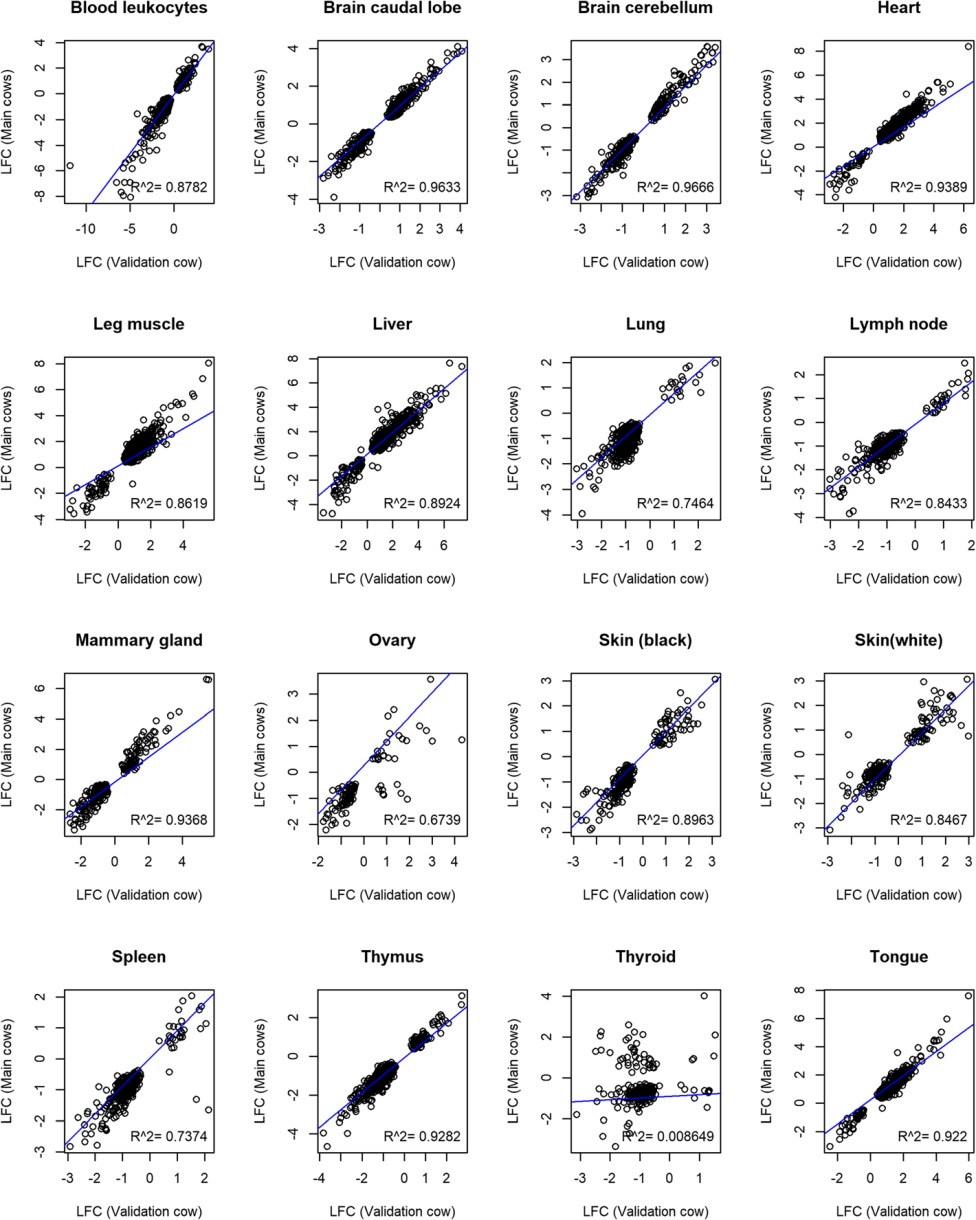
Scatterplot of log2 fold change values of differentially expressed mitochondrial protein genes from nuclear genome in the Main Cows against the Validation Cow

Firstly, both validation datasets confirmed the over-expression of MP genes in heart and skeletal muscles, and under-expression in blood leukocytes as in the adult tissues of the Main Cows dataset (Additional file 11-18). Further, expression of MP genes within tissues, as indicated by LFC values between Main Cows and Validation Cow (Fig. 5), were highly correlated (R^2^ 0.67–0.96) except for thyroid (R^2^ 0.01). Similarly, the correlation of LFC values between Main Cows and Validation Sheep was high (R^2^ 0.6–0.87), except for mammary and lungs (R^2^ 0.36, 0.34) (Additional file 19). We investigated the poor correlation of gene expression in thyroid between the Main Cows and the Validation Cow. At least 35 DE *Nu*MP genes in common between the datasets were expressed in opposite directions. These genes were mainly enriched for metabolic pathways, pyruvate metabolism and synthesis of antibiotics. Interestingly, the expression of *Nu*MP genes between Validation Cow and Validation Sheep were moderately correlated including thyroid (R^2^ 0.59) except for lung and mammary tissues (Additional file 20).

Secondly, the *‘either all over-expression or all under-expression*’ of DE *Mt*MP genes within tissues was supported by findings from both validation datasets. Further, the expression of *Mt*MP genes in the direction of the dominant DE *Nu*MP genes also remained evident across datasets.

Thirdly, the co-expression of *Mt*MP and *Nu*MP genes in a cluster were reproduced in the Validation Cow (Additional file 21), and to some extent in Validation Sheep (Additional file 22). The co-expression of MP genes in the *Nu*MP-*Mt*MP cluster in the Validation Cow was more than expected by random chance (χ^2^= 207.847, *p* < 0.01) showing that the enrichment of the cluster for co-expression of MP genes.

**Fig. 6:**
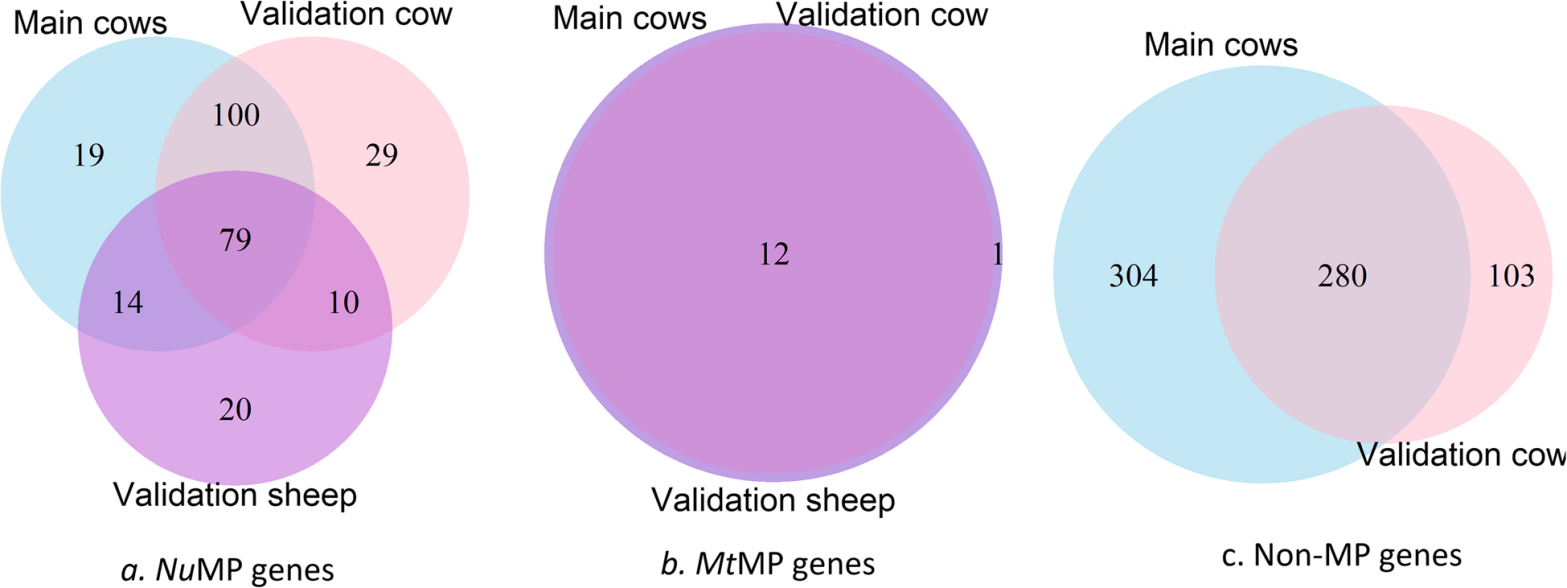
Venn diagram showing the number of genes in common among the *Nu*MP-*Mt*MP co-expression clusters. a. Mitochondrial protein genes encoded by the nuclear genome (*Nu*MP genes), b. Mitochondrial protein genes encoded by the mitochondrial genome (*Mt*MP genes) and c. non-mitochondrial protein genes from nuclear genomes in common between the Main Cows and Validation Cow

Finally, the overlap of genes in *Nu*MP-*Mt*MP clusters across the Main Cow and validation datasets was higher than would be expected if genes were randomly allocated to clusters. In particular, the occurrence of *Mt*MP genes were almost coincidental (13/13) between cow datasets and 12/13 genes in common between cow and sheep datasets. Similarly, a considerable proportion of *Nu*MP genes and also non-mitochondrial protein genes, were in common across datasets (Fig. 6).

## Discussion

This study described and validated specific patterns of differential expression for over 1000 mitochondrial protein genes, encoded by the nuclear and mitochondrial genomes, in bovine across different tissues. The study also presented strong evidence of co-expression between *Nu*MP and *Mt*MP genes.

### Differential expression of mitochondrial protein genes

#### Overexpression of MP gene in more metabolically active tissues

The observed patterns of differential expression of MP genes within tissue, where the proportion of DE MP genes exceeded 40%, appears to correlate with the known metabolic demand of tissues. MP genes were over-expressed in tissues with high reported metabolic demand (heart, skeletal muscles, tongue, and kidney cortex: Table 4), and under-expressed in tissues with low reported energy demand (adipose tissue and blood leukocytes: Table 4). In humans, about 60–70% of the total resting energy expenditure is accounted for by kidney, brain, liver, and heart, which altogether constitute less than 6% of adult body weight, whereas skeletal muscle (40–50% of body weight) accounts for 20–30% of resting energy expenditure [4, 39], which altogether account up to 80% of energy expenditure.

**Table 4:** Specific metabolic rates of organs and tissues across species (kcal/kg/day)

Species	Heart	Kidney	Brain	Liver	Skeletal muscle^a^	Adipose	Reference
Cattle	429	412	185	130	–	–	[36]
Sheep	588	496	255	200	–	–	[36]
Human	440	440	240	200	10–15	4.5	[4, 38]

^a^Rate for resting muscle

The heart meets almost the entire energy demand through the mitochondrial OXPHOS pathway (95%) [40]. A higher expression of selected *Mt*MP genes (*ND1, ND5, ATP6, CYTB*) were reported in the heart compared to other tissues (brain, kidney, liver and skeletal muscle) in mice [41], which supports heart as the tissue with the highest *Mt*MP gene expression. Similarly, skeletal muscles, which has low resting energy demand, are capable of spiking by almost 1000-fold during short intensive exercise [42, 43]. OXPHOS is highlighted as an important pathway for generating energy during the exercise/muscular activity in both short intensive as well as prolonged exercise [44]. The observed higher expression of MP genes in the tongue seems likely because the tongue is a muscular organ. Furthermore, results from the heart and skeletal muscles group reinforced the importance of OXPHOS and metabolic pathways in energy metabolism in these tissues.

A high expression of MP genes specifically in kidney cortices may be attributed to energy generation taking place at the proximal and distal convoluted tubules, which are also the site for active reabsorption of metabolites [45, 46]. In kidney and liver, the enrichment for metabolic pathways but not for OXPHOS, despite their high energy demand, is suggestive of dominance of non-OXPHOS pathways in energy metabolism.

#### Tissues with under-expression of mitochondrial protein genes

Among the tissues with under-expression of MP genes, only adipose tissue in human has a published metabolic rate. In keeping with our observed low MP gene expression in adipose, the metabolic rate of human adipose tissues was low (3.2–4.6 kcal/kg/day) [38]. On the other hand, leukocytes (and other tissues with under-expression for MP) have mainly non-energy related mitochondrial functions, such as redox signalling and controlling apoptosis [47], which, in part, could explain the incidence of under-expression of MP genes in blood leukocytes. Further, results from the analysis of group of tissues showing MP gene under-expression revealed a low number of DE genes in common across these tissues and no enrichment for energy pathways support a diminished role of mitochondrial energy function in leukocytes.

As for the adult cows, the highest expression of MP genes in the foetal heart tissue was expected considering the early foetal development and establishment of the heartbeat occurs as early as 3 weeks in the bovine foetus. In contrast to adult cows, the low expression of MP genes in foetal leg muscle was likely attributable to only partial development and non-functionality of the muscle. Skeletal muscle development, mainly secondary myogenesis, is initiated in the foetal stage from 9 weeks post-fertilization to parturition [48] and our foetal calves were around 16 weeks old. Generally, the remaining foetal tissues measured in this study are reported to be functionally inert in the foetal development stage, including lungs [49] and explains the under-expression of the MP genes.

In general, the expression profiles of MP genes in a tissue were consistent as indicated by clustering of same tissue of two or more animals within the dataset. Nonetheless, some tissues were exceptions, including foetal livers may be attributed to the sampling and cellular heterogeneity of the samples because each cell type may have a specific expression profile.

To sum up, the expression of MP genes in this study concurs with the energy demand of tissues (where known) implying that the increased energy demand may be met through increased expression of MP genes. Furthermore, previous studies report that there is a specific correlation between mRNA and protein quantity across tissues [50, 51].

### Mitochondrial genome encoded mitochondrial protein (*Mt* MP) gene expression

Besides, energy demand in tissues as the basis of increased transcription rates of MP genes, high *Mt*MP gene expression could also result from increased mitochondrial genome copy numbers. Mitochondrial DNA copy number differs considerably across tissue types, but remains closely regulated within a tissue type [23]. Studies in humans indicate that mitochondrial genome copy numbers are aligned with tissue energy demands: for example heart, skeletal muscle, omental fat, and blood leukocytes had 6970, 3650, 400–600 and 91 copies per diploid nuclear genome respectively [52–54]. Studies comparing copy number and gene expression of all *Mt*MP genes across tissues are scarce. A study in striated muscles (cardiac, type 1 skeletal muscle and type 2 skeletal muscles) of rabbit [55] demonstrated that the expression of *Mt*MP gene (*CYTB*) was proportional to mitochondrial copy number. Thus, it is plausible that the varying gene expression (indicating energy requirements) of tissue types are modulated through their mitochondrial DNA copy number.

#### Direction of expression of differentially expressed mitochondrial protein genes

There were two interesting aspects of the direction of DE *Mt*MP genes; first, the ‘*all over-expression or all under-expression*’ of DE *Mt*MP genes within tissues and second, the directional consistency of DE *Mt*MP genes expression in the dominant direction of DE *Nu*MP genes. The first phenomenon of occurrence of DE *Mt*MP genes in single direction has not been previously reported to the best of our knowledge. A possible explanation of this phenomenon rests in the mechanism of transcription because the entire mitochondrial genome is transcribed as a near-complete polycistronic unit [56, 57], so that almost all mitochondrial genes are transcribed as one unit. The initiation of transcription, particularly at *HSP2* promotor site on the mitochondrial genome generates a near-complete polycistronic unit [58]. The second trend showing the common direction of DE *Mt*MP and *Nu*MP genes was observed in all tissues exhibiting significant DE of *Mt*MP genes. This suggests DE *Nu*MP and *Mt*MP in these tissues are co-regulated.

### Co-expression of mitochondrial protein genes

The co-expression of mitochondrial protein genes was a prominent finding in the current study. Co-expression of MP gene in *Mt*MP-*Nu*MP cluster was further tested to be non-random and non-random co-expression of genes are previously reported across species [30]. Further, the significant enrichment of *Nu*MP-*Mt*MP co-expression cluster for OXPHOS and metabolic pathway supports co-functional co-expression of genes [29, 59]. Similarly, results from MP gene expression study in humans showed a significant correlation between *Mt*MP and *Nu*MP gene expression within tissues [60], suggesting close coordination between nuclear and mitochondrial genomes in relation to energy demand. The functional enrichment of our *Nu*MP-*Mt*MP cluster for the OXPHOS pathway and non-MP genes in the cluster for heart myopathies, contraction and signalling, emphasize their role in energy metabolism and supporting systems. The *Nu*MP cluster was enriched for metabolic pathways which is another important energy metabolism component of mitochondria.

The investigation of involvement of TAD on the co-expression demonstrated that the co-expressed genes in *Nu*MP-*Mt*MP cluster occurring in two or more within a TAD compared to the random sample. This indicated the potential role of TADs in co-expression of mitochondrial protein gene in our study. As such, the intra-TAD gene co-expression was not different from random for most chromosomes in another study [61].

### Validation

Overall, there were high correlation and consistency evident in the expression (differential expression and co-expression) of mitochondrial protein genes in tissue across the datasets. However, we have not considered for the physiological states, number of tissues sampled, and sequencing platforms employed in our validation study. Firstly, a notable difference in expression profile of *Nu*MP genes in the thyroid between the Main Cows and the Validation Cow, and Main Cows and Validation Sheep is potentially related to pregnancy of the Main Cows, as the Validation Cow and the Validation Sheep were not pregnant. The activity of thyroid and thyroid hormone synthesis are reportedly increased during pregnancy in human [62] and thyroid hormones are known to regulate metabolism [63]. The interaction of thyroid and MP function in metabolism is an area of interest for further investigation but beyond the scope of current work. Secondly, we based the differential gene expression of a gene in tissue to the mean expression across all other tissues where both the number of tissues and tissue types were not completely identical across the datasets (29 tissues in Main Cows, 18 tissues in Validation Cow and 15 tissues in Validation Sheep). Thereby, expression in tissue across the datasets has been compared to the mean expression of different sample sizes, which might vary across the datasets. Thirdly, the sequencing platforms used were different for each dataset: the Main Cows dataset were sequenced on HiSeq™ 3000 (Illumina), the Validation Cow was sequenced on HiSeq^TM^2000 sequencer (Illumina) and Validation Sheep were sequenced on Illumina HiSeq^TM^ 2500.

## Conclusions

Mitochondrial protein genes were differentially expressed across tissues. Tissues with high energy demand showed over-expression and under-expression was observed in tissues with low energy requirements, which suggests a link between mitochondrial protein gene expression and the energy demand of each tissue. Furthermore, mitochondrial protein genes from both genomes (*Nu*MP and *Mt*MP) were significantly co-expressed and enriched for co-functionality. This implies that it is necessary to consider mitochondrial protein genes from both genomes in studies related to mitochondrial function. Mitochondrial protein gene expression analysis may be extrapolated to production traits such as feed efficiency, heat tolerance, adaptability to cold climate, to further elucidate their role in relation to energy metabolism.

## Methods

### Data

The standard best practice recommendations for RNA-seq is at least three samples of each tissue (from different individuals) [64]. This study utilized RNAseq from three cows; two Holstein cows and their foetuses, and one Holstein cow from a previous study [27]. As the cows in the two datasets were physiologically different due pregnancy status and also used different sequencing platforms, we analysed them separately and the results from the two cows dataset (Main Cows) was validated in the one cow dataset (Validation Cow). Further, gene expression patterns in cattle were validated in a sheep dataset previously published [65], which is a closely related species (Validation Sheep) [37]. The Main Cows dataset had RNAseq from 29 tissues from two adult cows and six tissues from two 16 weeks old foetuses. The Validation Cow data consisted of RNAseq reads from 18 tissues, and the Validation Sheep data were gene expression counts for a subset of tissues (15 tissue types) of three Texel x Blackface adult females. The tissue-specific gene expression patterns in the Main Cows dataset were validated using the validation datasets.

### Ethics, animals and tissue sampling

The ethical approval, including the permission to euthanise the animals of the Main Cows datasets were obtained from the Department of Jobs, Precincts and Regions Ethics Committee (Application No. 2014–23). Two lactating and pregnant Holstein cows and their two foetuses at 16 weeks of gestation representing a comparable physiological status from the Agriculture Victoria Research dairy herd at Ellinbank, Victoria, Australia (38°14′ S, 145°56′ E) were chosen for the study. The cows were offered 6 kg of wheat per day with perennial ryegrass pasture grazed in the paddock, supplemented with pasture silage or hay where required. Both cows were born in 2006, 16 weeks pregnant and were sampled on day 205 and 173 of their lactation (cow 2181 and 6819, respectively). Cow 2181 had a male foetus (2181F), and cow 6819 had a female foetus (6819F). Both foetuses were from the same sire (half-sibs).

Blood samples were drawn from the coccygeal vein by venipuncture before euthanasia and processed following the blood fractionation and white blood cell stabilization protocols of the RiboPure™ blood kit (Ambion by Life Technologies). Other tissues were sampled following euthanasia of the animals. The cows were euthanised individually by a trained veterinarian and not within line of sight of another deceased animal to minimise stress. The cow was restrained in a crush and given an intravenous injection of 600 mg of xylazine IV adequate to cause moderate sedation. The cow was immediately released from the crush, and once the sedation had taken effect and the cow was sitting down, 300 mg of ketamine was given intravenously. Once the cow laid down, 1 l of 25% magnesium sulphate solution was administered intravenously until pronounced deceased by the veterinarian. Once pronounced dead, all tissue types were dissected from the animal. Connective tissue was removed, and the samples were dissected into 1 cm squares, sealed in a 5 ml tube and flash-frozen in liquid nitrogen. Subcutaneous fat was sampled from the rib region. Blood (on ice) and tissues samples (in liquid nitrogen canisters) were then moved to the main laboratory and stored at − 80 °C. The metadata and RNAseq reads for all 40 tissues are available at EMBL-EBI European Nucleotide Archive (ENA) under study accession ERP118133. For this study, we generated data for 35 samples (29 tissues from adult cows and six from the foetuses) (Table 5).

**Table 5:** List of 35 organ-tissue sampled from the two adult cows and two foetuses in the Main Cows dataset

Tissues/organs	Tissues/organs	Tissues/organs
Adrenal gland cortex (Adrenal cortex)	Lung	Spleen
Adrenal gland medulla (Adrenal medulla)	Mammary gland (Mammary)	Thymus
Omental fat pad (Omental fat)	Heart	Thyroid gland (Thyroid)
Subcutaneous fat	Brain cerebellum	Tongue
Kidney cortex	Brain stem	Blood leukocytes (Leukocytes)
Kidney medulla	Brain caudal lobe	
Longissimus thoracic muscle (Loin muscle)	Pituitary gland	Foetal brain
Semimembranosus muscle (Leg muscle)	Placenta	Foetal kidney
Intercostal muscle (Rib muscle)	Liver	Foetal lung
Latissimus dorsi muscle (Msub)	Skin black	Foetal heart
Mediastinal lymph node (Mediastinal LN)	Skin white	Foetal liver
Lymph node	Ovary	Foetal leg muscle

### RNA extraction and sequencing

RNA from blood leukocytes was extracted using the RiboPure Blood Kit (Ambion) according to the manufacturer instructions. For tissues, 100 mg of tissue was ground in a TissueLyserII (Qiagen) with liquid nitrogen, and then ~ 30 mg of ground tissue was used to extract RNA using Trizol (Invitrogen) following standard procedures. RNA was passed through a PureLink RNA Mini column (Qiagen) for clean-up and concentration and eluted in 30 μl RNase free water. RNA Integrity Numbers (RIN), which indicates the RNA quality, were determined using Agilent Tapestation (Agilent) and RNAseq libraries were prepared from all samples (Additional file 1) with RIN > 6 at which the 3′ bias level is at a minimum using the SureSelect Strand-Specific RNA Library Prep Kit (Agilent) as instructed by the manufacturer. Libraries were barcoded uniquely, assigned randomly to one of two pools and sequenced on a HiSeq™ 3000 (Illumina) in a 150-cycle paired-end run. One hundred and fifty bases paired-end reads were called with bcltofastq and output in fastq format. The quality of the libraries and alignment are as presented in Additional file 2. Poor-quality bases were filtered, and sequence reads trimmed using an in-house script. Bases with a quality score of < 20 were trimmed from the 3′ end of reads. Reads with a mean quality score < 20, > 3 N’s, or final length < 50 bases were not included. Only paired reads were retained for alignment.

### Read alignment and gene counting

For each library, paired-end reads were mapped to Ensembl bovine genome UMD3.1 reference [66] and annotated using STAR version 2.5.3ab [67]. Aligned reads were checked for quality using Qualimap 2 [68], and unique mapping reads for samples (Additional file 3). The R package featureCounts [69] was used to generate a count matrix of read counts per gene for every sample.

### Mitochondrial protein genes

Mitochondrial protein genes in the current study were based on the list of MP identified in humans, available in Mitocarta 2.0 [10]. The official gene names of 1158 MP genes were directly converted to bovine ensemble gene IDs using a gene ID conversion function in the software DAVID (Database for Annotation, Visualization, and Integrated Discovery) version 6.8 [70, 71]. This translated into 1054 bovine MP ensemble gene IDs (1041 *Nu*MP and 13 *Mt*MP), which were used as the final list of MP genes for further analysis in this study (Additional file 4). Additionally, 24 non-protein coding genes from the mitochondrial genome (22 *tRNA*s and 2 *rRNA*s) were also included in the analysis. The mitochondrial protein gene expression profiles in tissues are expected to be similar across mammalian species because they share a very important mitochondrial function [72, 73].

### Differential gene expression analysis

The lowly expressed genes were filtered out using function filterByExpr of edgeR package for differential expression analysis in R [74]. Differential expression of genes was analysed using the glmQLFit function. A model was fitted to the data with a design matrix of an overall mean of gene expression counts across all other tissues as the intercept and tissue as a fixed effect, i.e. differential expression is relative to the average expression of the same gene across all other tissues. The glmQLTest method was used to identify DE genes, specifically up or down expressed. A list of DE genes, along with their fold changes, was generated and summarized for each tissue. A gene was considered as differentially expressed (DE) in tissue if its expression was significantly higher than the mean expression of same gene across all other tissues (i.e. ≥ |0.6| log_2_ fold changes (LFC) = 1.5-fold difference, FDR < 0.01). The sign + and - of LFC values of the DE gene was used to deduce the expression as either over-expression or under-expression respectively compared to the mean of expression across all other tissues. Further, log_2_CPM (counts per million) values of all *Nu*MP and *Mt*MP genes (including non-DE MP genes) across tissues were visualized as heatmap using R package pheatmap [75]. In addition, we looked at the number of DE MP genes by genome (i.e. *Nu*MP and *Mt*MP), their direction of expression and the proportion of DE genes to the total genes in category. The foetal tissues were analysed separately following the procedures implemented for the adult cows.

### Co-expression network analysis across tissues

The functionally associated genes tend to be co-expressed, and this is used to infer novel function as well as to identify candidate genes in diseases and their prediction [28]. To study the co-expression pattern in tissues, we used a similarity network based on a Pearson correlation coefficient of gene expression (>|0.95|) of adult cows in the Main Cows dataset, executed using a plugin ExpressionCorrelation [76] in Cytoscape 3.6.1 [77], to construct gene co-expression clusters. We analysed the co-expression cluster involving MP genes for;

i. biological significance of the cluster using functional enrichment analysis and composition of the genes,
ii. whether the co-expression of *Nu*MP genes in *Nu*MP-*Mt*MP cluster was greater than random expectations using Chi-square goodness of fit (χ^2^);

$$ {\chi}^2=\sum \frac{{\left({O}_i-{E}_i\right)}^2}{E_i} $$

Where,*O*_*i*_ = observed frequency of genes _*(i)*_ in the *Nu*MP-*Mt*MP cluster (_*i*_ = *Nu*MP, Non-*NuMP* gene).

*E*_*i*_ = expected frequency of genes _*(i)*_ from the overall clusters (_*i*_ = *Nu*MP, Non-*NuMP* gene)

iii. the effect of TAD in co-expression of *Nu*MP genes in *Nu*MP-*Mt*MP cluster considering TAD as one of the several factors potentially influencing the co-expression of small group of genes. Briefly, we mapped the co-expressed bovine genes across the clusters (3643) to the putative bovine TADs derived from the IMR90hg18 [78] and 3022 genes mapped to 1286 TADs. Similarly, within the *Nu*MP-*Mt*MP cluster, 651 of 813 co-expressed genes mapped to 484 TADs. Of this, 282 co-expressed genes were distributed in groups of 2 or more per TAD. We compared this to averages from 100 random samples of 651 genes from TAD mapping genes across all clusters (3022). The averages for number of TADs of the random samples and genes found in group of 2 or more within a TAD were 472 (±10) and 116 (±10) respectively.

### Functional enrichment analysis

The DAVID software was used to investigate the functional enrichment of differentially expressed genes within a tissue and co-expressed genes across tissues: up to 3000 genes (maximum permissible in DAVID) were selected and analysed for over-representation in KEGG (Kyoto Encyclopedia of Genes and Genomes) pathways [79]. Up to top 10 pathways with adjusted *p* < 1e-5 are discussed in this study.

### Validation

The patterns of MP gene expression in tissues of Main Cows were validated using two previously published datasets: a lactating Holstein cow (2 years old, 65 days in milk) with 18 tissues (additional file 17) [27] (i.e. Validation Cow); and three adult female Texel x Scottish Blackface sheep from the sheep gene expression atlas project [37], which were aged about 2 years and locally (Scotland) acquired (i.e. Validation Sheep). Depending on the number of tissues in common with cattle datasets, 15 tissues were chosen from the sheep study (Additional file 18). The Validation Cow was analysed separately due to its difference in physiological status compared to the Main Cows dataset. The RNAseq reads of the Validation Cow were processed, aligned, gene counts generated and analysed following the protocols for Main Cows. Similarly for sheep, the raw gene counts [65] were normalized and subjected to standard processing and analyses for differential expression and co-expression. In sheep, 823 MP genes were identified as overlapping the Mitocarta 2.0 Human database, using the same approach as in cattle (Additional file 5). The pattern of MP gene expression across tissues was visualized with a heatmap and co-expression networks as described for Main Cows. One of the purposes of validation was to look at the consistency of gene expression patterns across datasets. To evaluate the consistency of differential expression of MP gene expression in a tissue across the datasets, a scatterplot of the LFC values of DE *Nu*MP genes (in common between the datasets) and their coefficient of determination (R^2^) was used to indicate correlation between datasets. For consistency in co-expression of MP genes, the *Nu*MP-*Mt*MP co-expression cluster was further examined for the composition and commonality of genes among the datasets.

## Supplementary information

**Additional file 1: Table S1.** Average RIN by tissue types.

**Additional file 2: Table S2.** Quality of library preparation.

**Additional file 3: Table S3.** Read alignment quality check.

**Additional file 4: Table S4.** List of Mitochondrial protein genes derived from Mitocarta in cattle.

**Additional file 5: Table S5.** List of Mitochondrial protein genes derived from Mitocarta in Sheep.

**Additional file 6: Table S6.** Number of differentially expressed (DE) genes by gene categories averaged for two foetuses in the Main Cows.

**Additional file 7: Figure S1.** Heatmap of expression of nuclear genome encoded mitochondrial protein (*Nu*MP) in tissues of foetuses 6819F and 2181F in the Main Cows.

**Additional file 8: Figure S2.** Heatmap of mitochondrial genome encoded mitochondrial protein (*Mt*MP) genes in tissues of foetuses 6819F and 2181F in the Main Cows.

**Additional file 9: Table S7.** List of non-mitochondrial protein (Non-MP) genes clustering with the mitochondrial protein genes in cluster I (*Nu*MP-*Mt*MP cluster) in the Main Cows.

**Additional file 10: Table S8.** KEGG pathway enrichment of the non-mitochondrial protein (Non-MP) genes in *Nu*MP-*Mt*MP cluster in the Main Cows

**Additional file 11: Figure S3*.*** The proportion of differentially expressed gene in each gene category in 18 tissues in a Validation Cow (All=All genes encoded by nuclear and mitochondrial genome, Nu=Mitochondrial protein genes encoded by nuclear genome (*Nu*MP), Mt=Mitochondrial protein genes encoded by mitochondrial genome (*Mt*MP).

**Additional file 12: Figure S4.** Heatmap of expression of nuclear genome encoded mitochondrial (*Nu*MP) gene in the Validation Cow.

**Additional file 13: Figure S5.** Heatmap of expression of mitochondrial genome encoded mitochondrial protein (*Mt*MP) genes in the Validation Cow.

**Additional file 14: Figure S6.** The proportion of differentially expressed gene in each gene category and direction of gene regulation in 15 tissues in the Validation Sheep (All=All genes encoded by nuclear and mitochondrial genome, Nu=Mitochondrial protein genes encoded by nuclear genome (*Nu*MP), Mt=Mitochondrial protein genes encoded by mitochondrial genome (*Mt*MP).

**Additional file 15: Figure S7.** Heatmap of nuclear genome encoded mitochondrial protein genes (*Nu*MP) in the Validation Sheep (three adults Texel x Blackface female sheep AF1, AF2, and AF3).

**Additional file 16: Figure S8.** Heatmap of mitochondrial genome encoded mitochondrial protein genes (*Mt*MP) genes in Validation Sheep (three adults Texel x Blackface females AF1, AF2, and AF3).

**Additional file 17: Table S9.** Number of differentially expressed gene (DEG) s and their direction in tissues by gene categories in the Validation Cow.

**Additional file 18: Table S10.** Number of differentially expressed gene (DEG) s and their direction in tissues by gene categories in the Validation Sheep.

**Additional file 19: Figure S9.** Scatter plot of log fold changes of the Main Cows against the log-fold changes of the Validation Sheep for mitochondrial protein gene expression in tissues.

**Additional file 20: Figure S10.** Scatter plot of log fold changes of the Validation Cow against the log-fold changes of the Validation Sheep for mitochondrial protein gene expression.

**Additional file 21: Figure S11.** Gene co-expression network constructed based similarity matrix computed using Person Correlation Co-efficient of gene expression at r > |0.95| across tissues of the Validation Cow.

**Additional file 22: Figure S12.** Gene co-expression network constructed based similarity matrix computed using Person correlation coefficient of gene expression at r > |0.95| across tissues of the Validation Sheep (three Texel x blackface adult female sheep AF1, AF2 and AF3).

## Data Availability

RNAseq datasets for two cows and their foetuses are available at EMBL EBI European Nucleotide Archive (ENA) under study accession ERP118133 at https://www.ebi.ac.uk/ena/data/search?query=ERP118133, and for Validation Cow in fastq format under study accession number ERP107617 at https://www.ebi.ac.uk/ena/data/search?query=ERP107617. The raw gene counts from three adult Texel X Blackface female 1, 2 and 3 are from the sheep gene expression atlas dataset available at https://doi.org/10.7488/ds/2616. The bovine reference genome UMD 3.1 is available at https://www.ncbi.nlm.nih.gov/assembly/GCA_000003055.5, and the list of human mitochondrial proteins (MitoCarta 2.0) is available at www.broadinstitute.org/pubs/MitoCarta. The data generated and analysed in our study supporting the conclusions of the article are included in the Additional files.

## Abbreviations

CPM: Counts Per Million
DAVID: Database for Annotation, Visualization, and Integrated Discovery
DE: Differentially expressed
EDTA: Ethylenediaminetetraacetic acid
FDR: False discovery rate
KEGG: Kyoto Encyclopedia of Genes and Genomes
LFC: Log2 fold change
MP: Mitochondrial proteins
MPG: Mitochondrial protein genes
*Mt*: Mitochondrial
*Mt*MP: Mitochondrial genome encoded mitochondrial proteins
NAFLD: Non-alcohol fatty liver disease
*Nu*MP: Nuclear genome encoded mitochondrial proteins
OXPHOS: Oxidative phosphorylation
PBS: Phosphate Buffer Saline
RIN: RNA integrity number
RNA: Ribonucleic acid
rRNA: Ribosomal ribonucleic acid
TAD: Topologically associating domain
TCA: Tricarboxylic acid
tRNA: Transfer ribonucleic acid
