# Correction to: Expression of mitochondrial protein genes encoded by nuclear and mitochondrial genomes correlate with energy metabolism in dairy cattle

**DOI:** 10.1186/s12864-022-08404-z

**Published:** 2022-04-20

**Authors:** Jigme Dorji, Christy J. Vander Jagt, Josie B. Garner, Leah C. Marett, Brett A. Mason, Coralie M. Reich, Ruidong Xiang, Emily L. Clark, Benjamin G. Cocks, Amanda J. Chamberlain, Iona M. MacLeod, Hans D. Daetwyler

**Affiliations:** 1 School of Applied Systems Biology, La Trobe University, Bundoora, VIC 3083 Australia; 2 Agriculture Victoria, AgriBio, Centre for AgriBioscience, Bundoora, VIC 3083 Australia; 3 Agriculture Victoria, Ellinbank Dairy Centre, Ellinbank, VIC 3822 Australia; 4 Faculty of Veterinary & Agricultural Science, University of Melbourne, Parkville, VIC 3052 Australia; 5 The Roslin Institute and Royal (Dick) School of Veterinary Studies, University of Edinburgh, Edinburgh, Scotland, UK

**Correction to: BMC Genomics 21, 720 (2020)**

**https://doi.org/10.1186/s12864-020-07018-7**

Following publication of the original article [1], it was reported that Fig. 4 was missing several labels (I-IV) and that Additional files 1, 2, 3, 4, 5, 6, 9, 10, 17 and 18 were published in an incorrect order and Additional file 20 was published with an erroneous caption. The correct Fig. 4 and additional files are provided in this correction article, and the original article [1] has been updated.

**Fig. 4:**
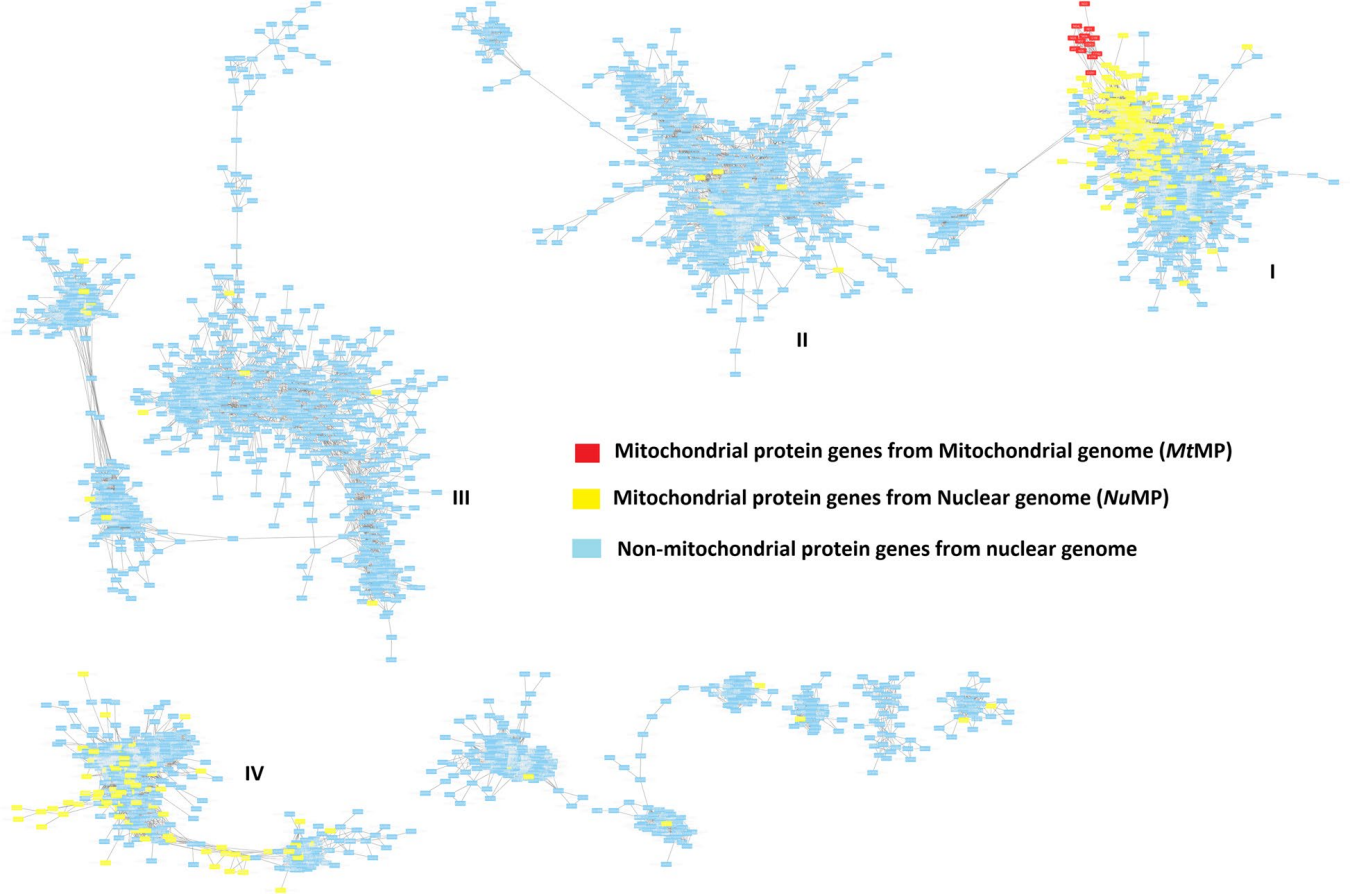
Gene co-expression network clusters across tissues in the Main Cows based on similarity matrix computed using Pearson correlations > |0.95|

## Supplementary Information

**Additional file 1: Table S1.** Tissue libraries and their RIN.

**Additional file 2: Table S2.** Quality of library preparation.

**Additional file 3: Table S3.** Read alignment quality check**.**

**Additional file 4: Table S4.** List of Mitochondrial protein genes derived from Mitocarta in cattle.

**Additional file 5: Table S5.** List of Mitochondrial protein genes derived from Mitocarta in Sheep.

**Additional file 6: Table S6.** Number of differentially expressed (DE) genes by gene categories averaged for two foetuses in the Main Cows.

**Additional file 9: Table S7.** List of non-mitochondrial protein (Non-MP) genes clustering with the mitochondrial protein genes in cluster I (NuMP-MtMP cluster) in the Main Cows.

**Additional file 10: Table S8.** KEGG pathway enrichment of the non-mitochondrial protein (Non-MP) genes in NuMP-MtMP cluster in the Main Cows

**Additional file 17: Table S9.** Number of differentially expressed gene (DEG) s and their direction in tissues by gene categories in the Validation Cow.

**Additional file 18: Table S10.** Number of differentially expressed gene (DEG) s and their direction in tissues by gene categories in the Validation Sheep.

**Additional file 20: Figure S10.** Scatter plot of log fold changes of the Validation Cow against the log-fold changes of the Validation Sheep for mitochondrial protein gene expression.
