# Supplementary figures and images for: Expression of mitochondrial protein genes encoded by nuclear and mitochondrial genomes correlate with energy metabolism in dairy cattle
Source: BMC Genomics. 2020 Oct 19;21:720. doi: 10.1186/s12864-020-07018-7 (PMC7574280; doi:10.1186/s12864-020-07018-7)

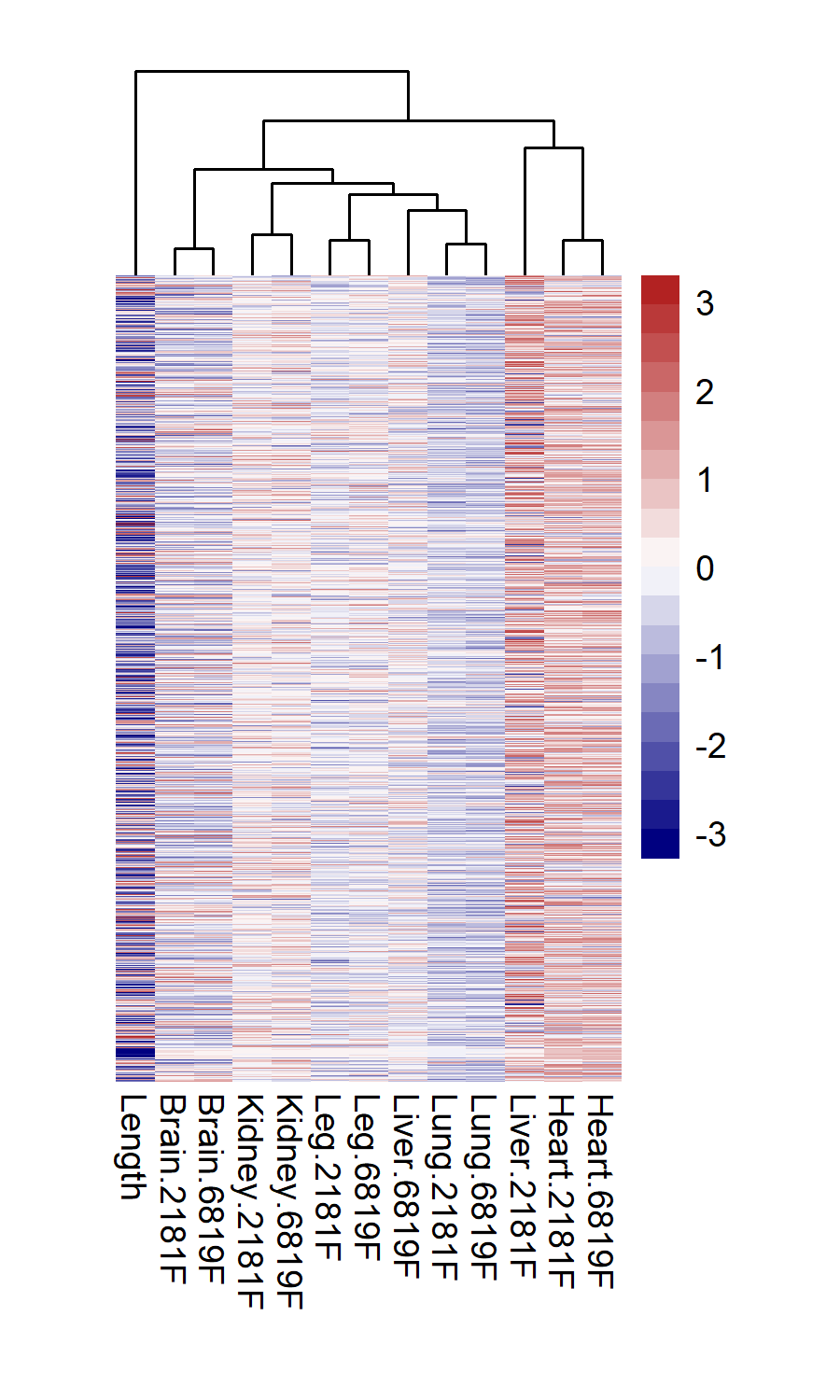

Supplement: Supplementary file 7 — Additional file 7: Figure S1. Heatmap of expression of nuclear genome encoded mitochondrial protein (NuMP) in tissues of foetuses 6819F and 2181F in the Main Cows. [file 12864_2020_7018_MOESM7_ESM.tiff]

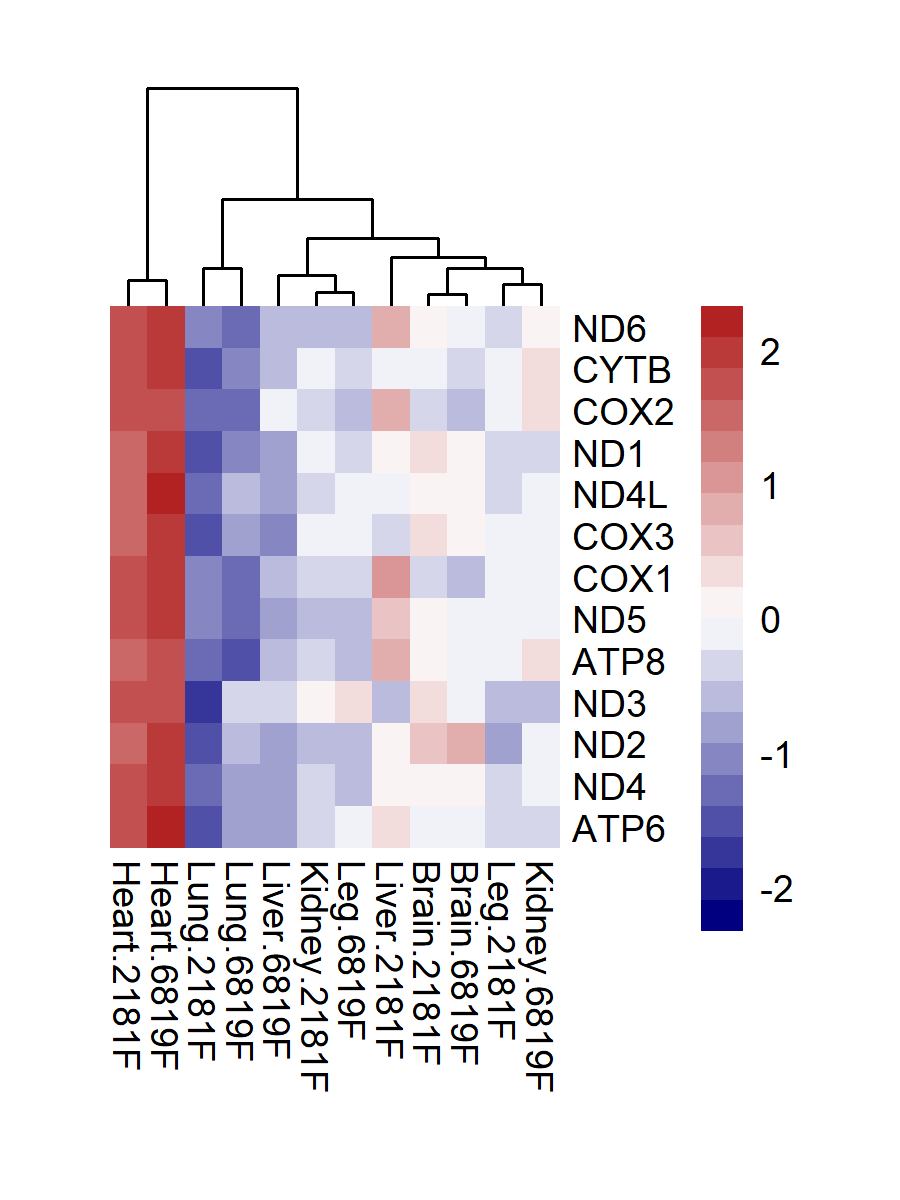

Supplement: Supplementary file 8 — Additional file 8: Figure S2. Heatmap of mitochondrial genome encoded mitochondrial protein (MtMP) genes in tissues of foetuses 6819F and 2181F in the Main Cows. [file 12864_2020_7018_MOESM8_ESM.tiff]

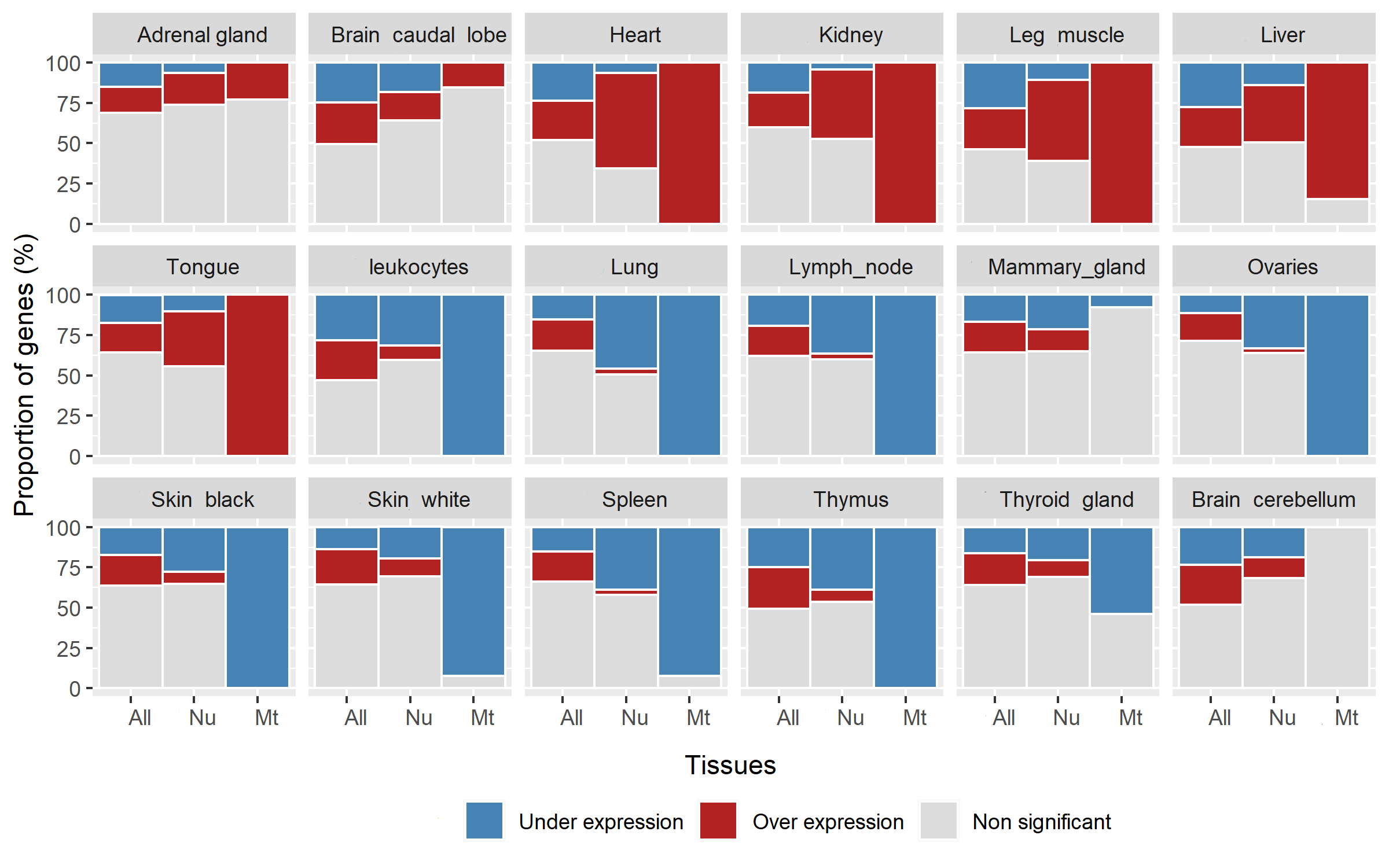

Supplement: Supplementary file 11 — Additional file 11: Figure S3. The proportion of differentially expressed gene in each gene category in 18 tissues in a Validation Cow (All=All genes encoded by nuclear and mitochondrial genome, Nu=Mitochondrial protein genes encoded by nuclear genome (NuMP), Mt=Mitochondrial protein genes encoded by mitochondrial genome (MtMP). [file 12864_2020_7018_MOESM11_ESM.tiff]

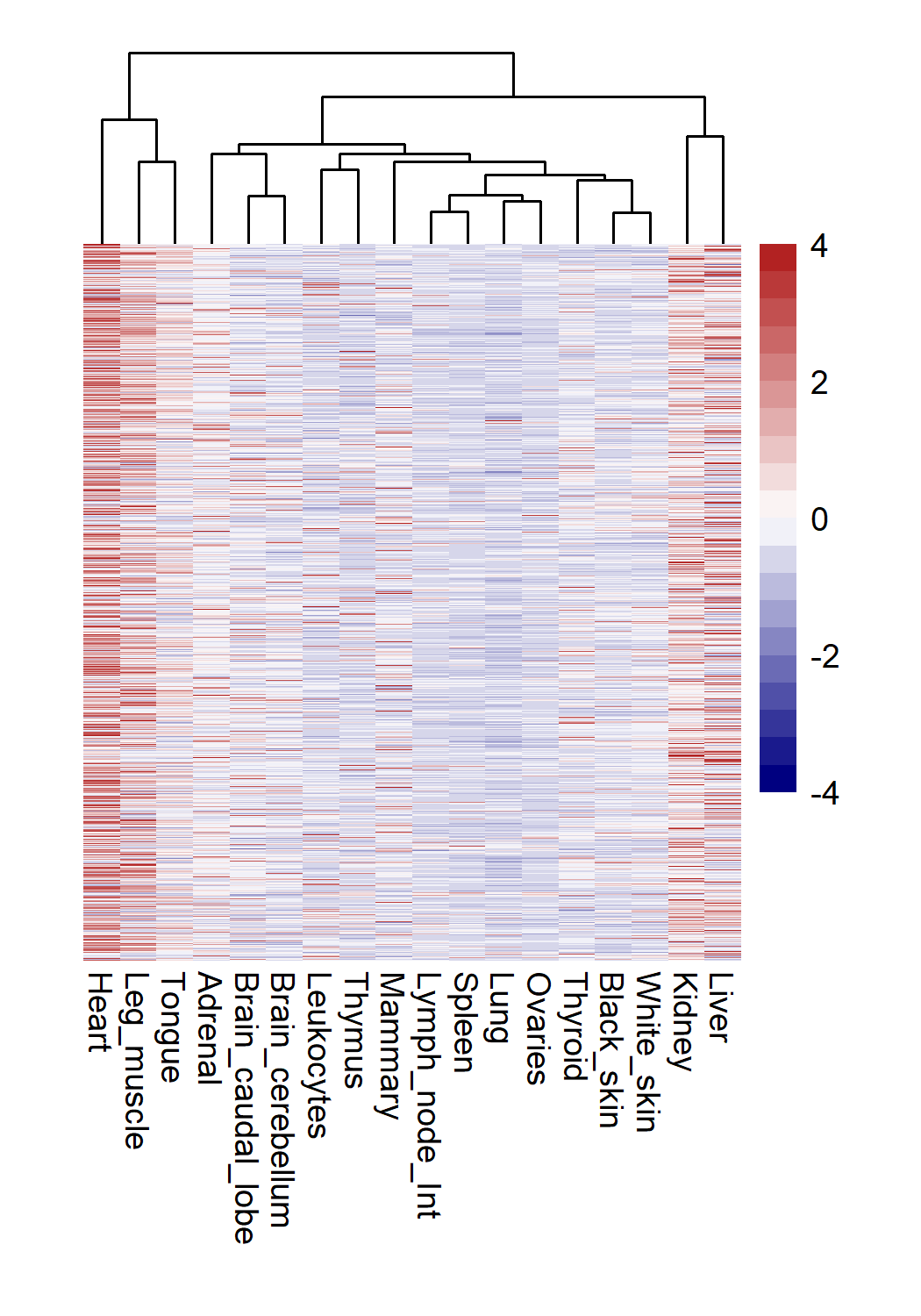

Supplement: Supplementary file 12 — Additional file 12: Figure S4. Heatmap of expression of nuclear genome encoded mitochondrial (NuMP) gene in the Validation Cow. [file 12864_2020_7018_MOESM12_ESM.tiff]

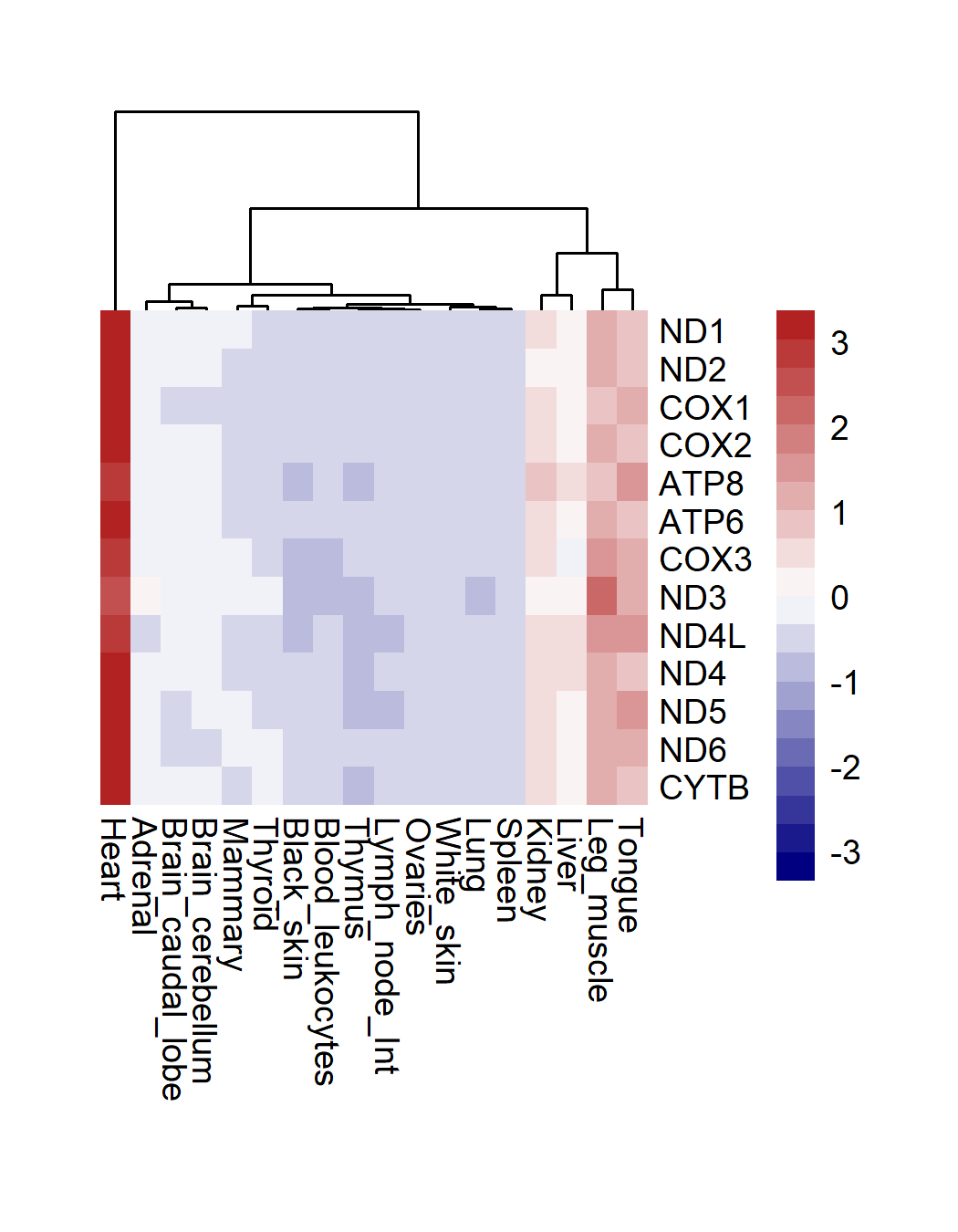

Supplement: Supplementary file 13 — Additional file 13: Figure S5. Heatmap of expression of mitochondrial genome encoded mitochondrial protein (MtMP) genes in the Validation Cow. [file 12864_2020_7018_MOESM13_ESM.tiff]

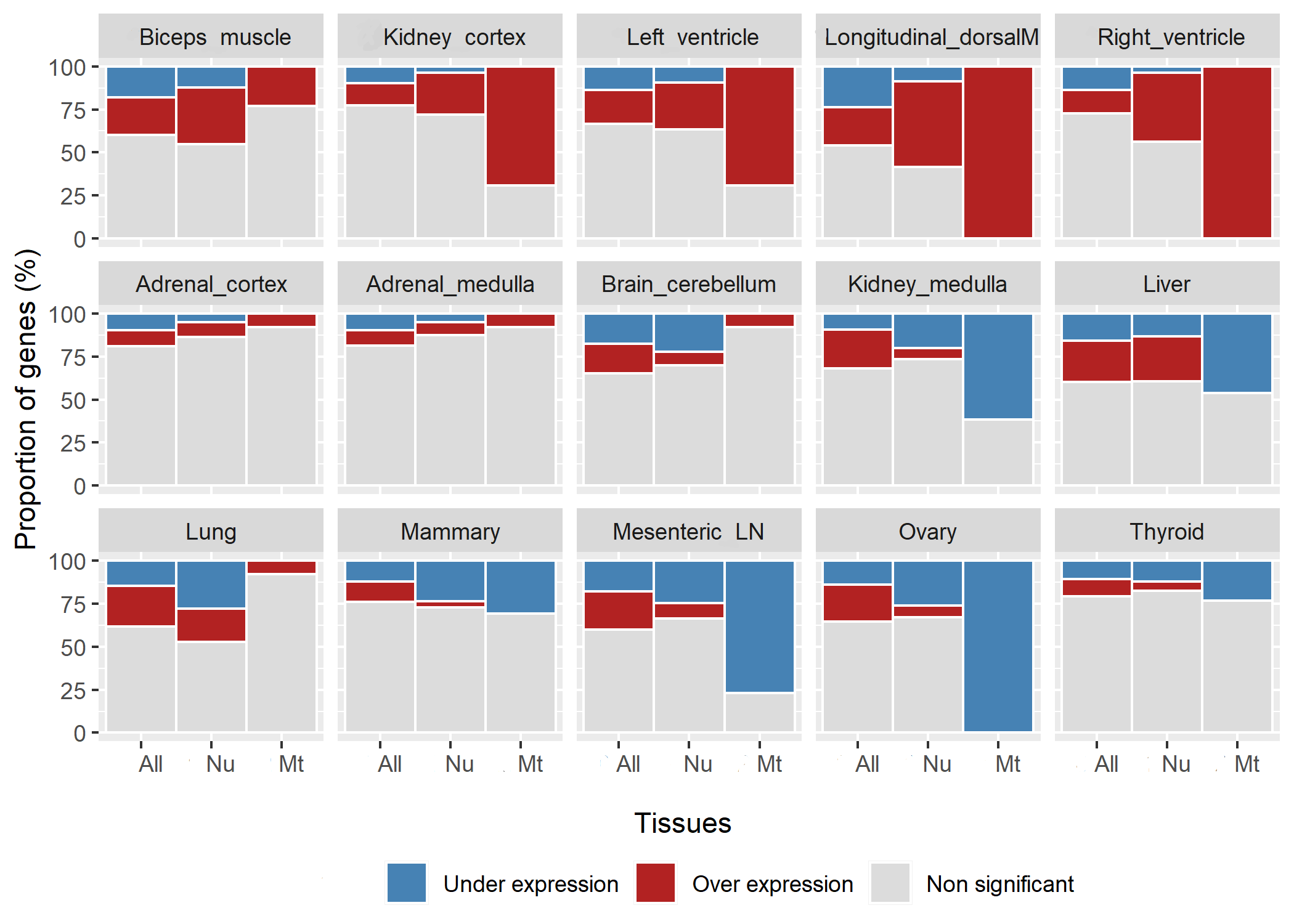

Supplement: Supplementary file 14 — Additional file 14: Figure S6. The proportion of differentially expressed gene in each gene category and direction of gene regulation in 15 tissues in the Validation Sheep (All=All genes encoded by nuclear and mitochondrial genome, Nu=Mitochondrial protein genes encoded by nuclear genome (NuMP), Mt=Mitochondrial protein genes encoded by mitochondrial genome (MtMP). [file 12864_2020_7018_MOESM14_ESM.tiff]

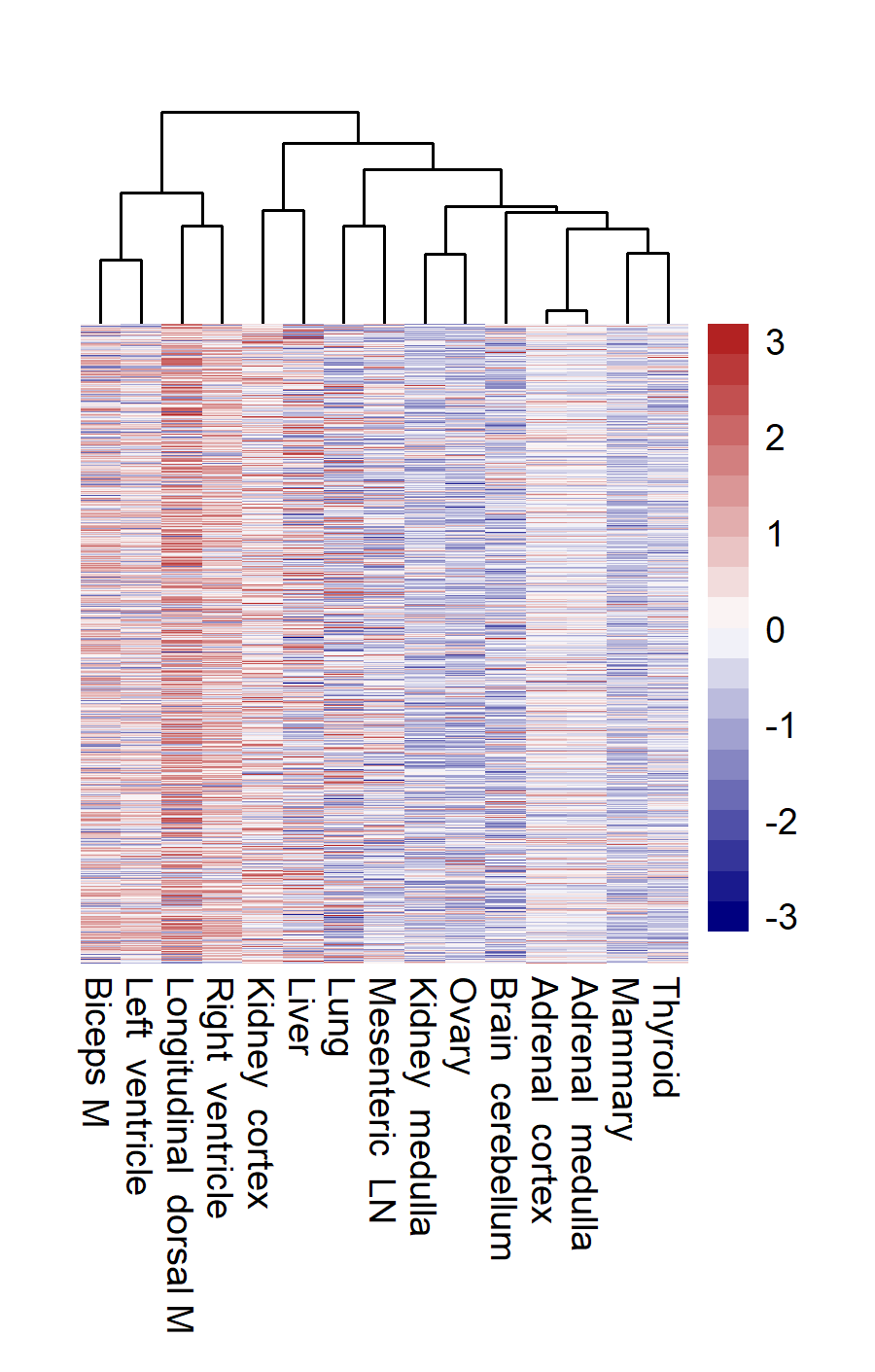

Supplement: Supplementary file 15 — Additional file 15: Figure S7. Heatmap of nuclear genome encoded mitochondrial protein genes (NuMP) in the Validation Sheep (three adults Texel x Blackface female sheep AF1, AF2, and AF3). [file 12864_2020_7018_MOESM15_ESM.tiff]

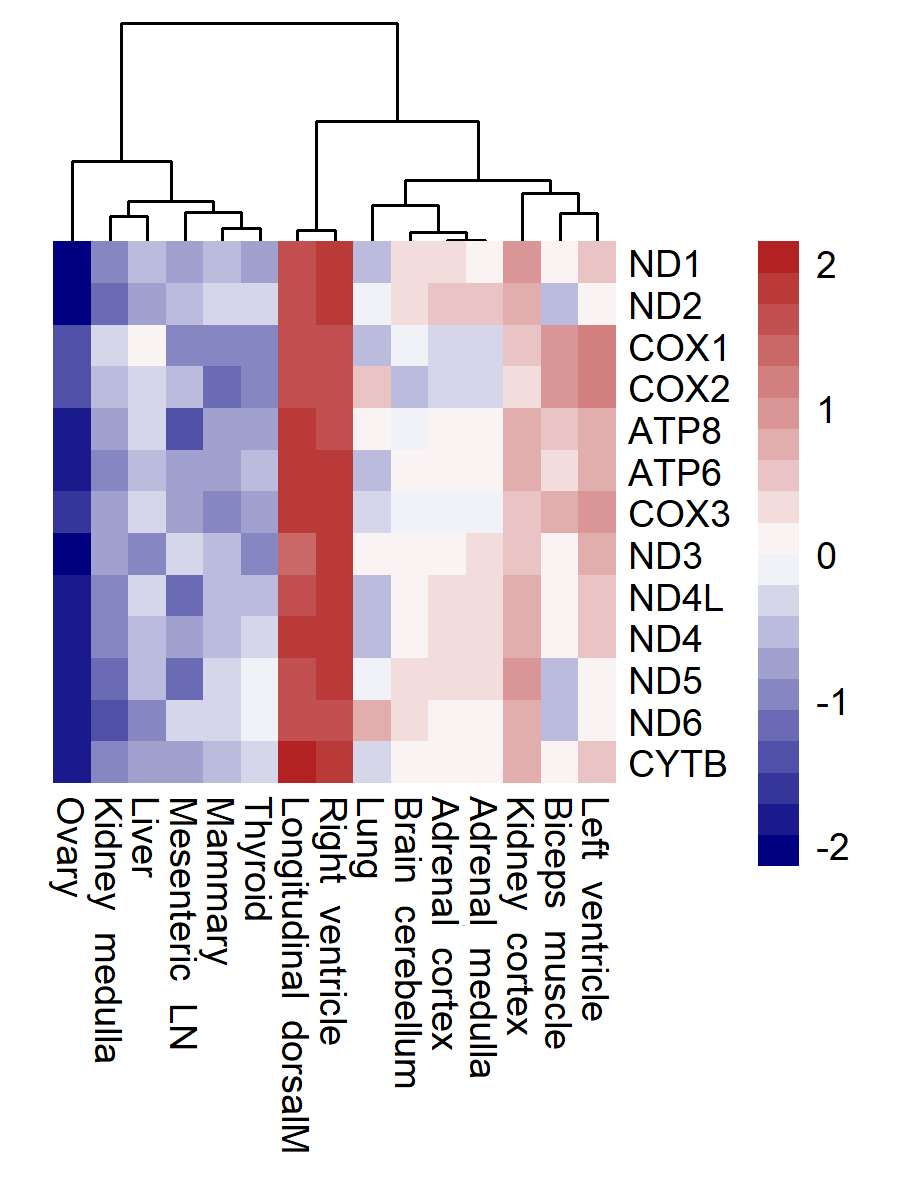

Supplement: Supplementary file 16 — Additional file 16: Figure S8. Heatmap of mitochondrial genome encoded mitochondrial protein genes (MtMP) genes in Validation Sheep (three adults Texel x Blackface females AF1, AF2, and AF3). [file 12864_2020_7018_MOESM16_ESM.tiff]

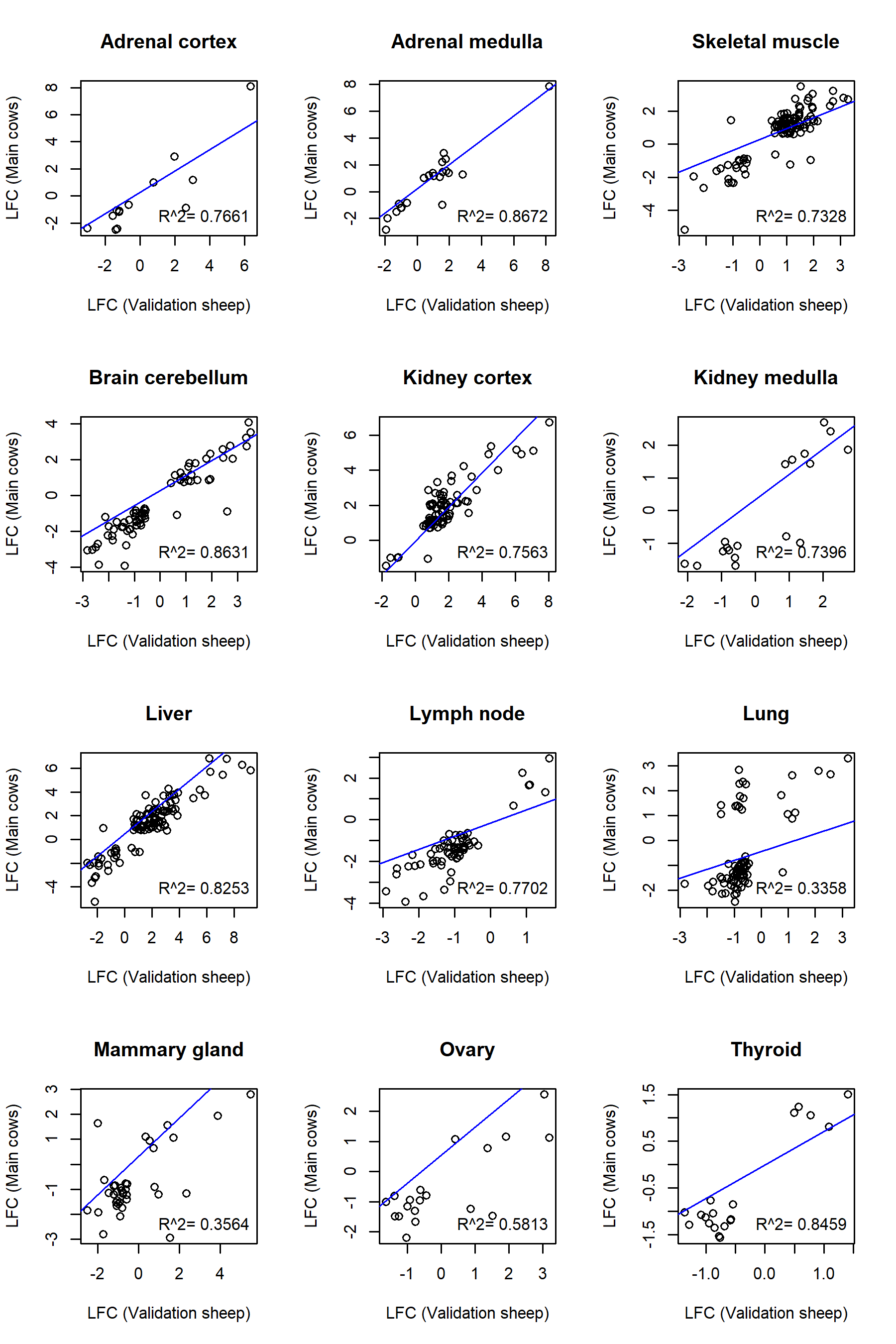

Supplement: Supplementary file 19 — Additional file 19: Figure S9. Scatter plot of log fold changes of the Main Cows against the log-fold changes of the Validation Sheep for mitochondrial protein gene expression in tissues. [file 12864_2020_7018_MOESM19_ESM.tiff]

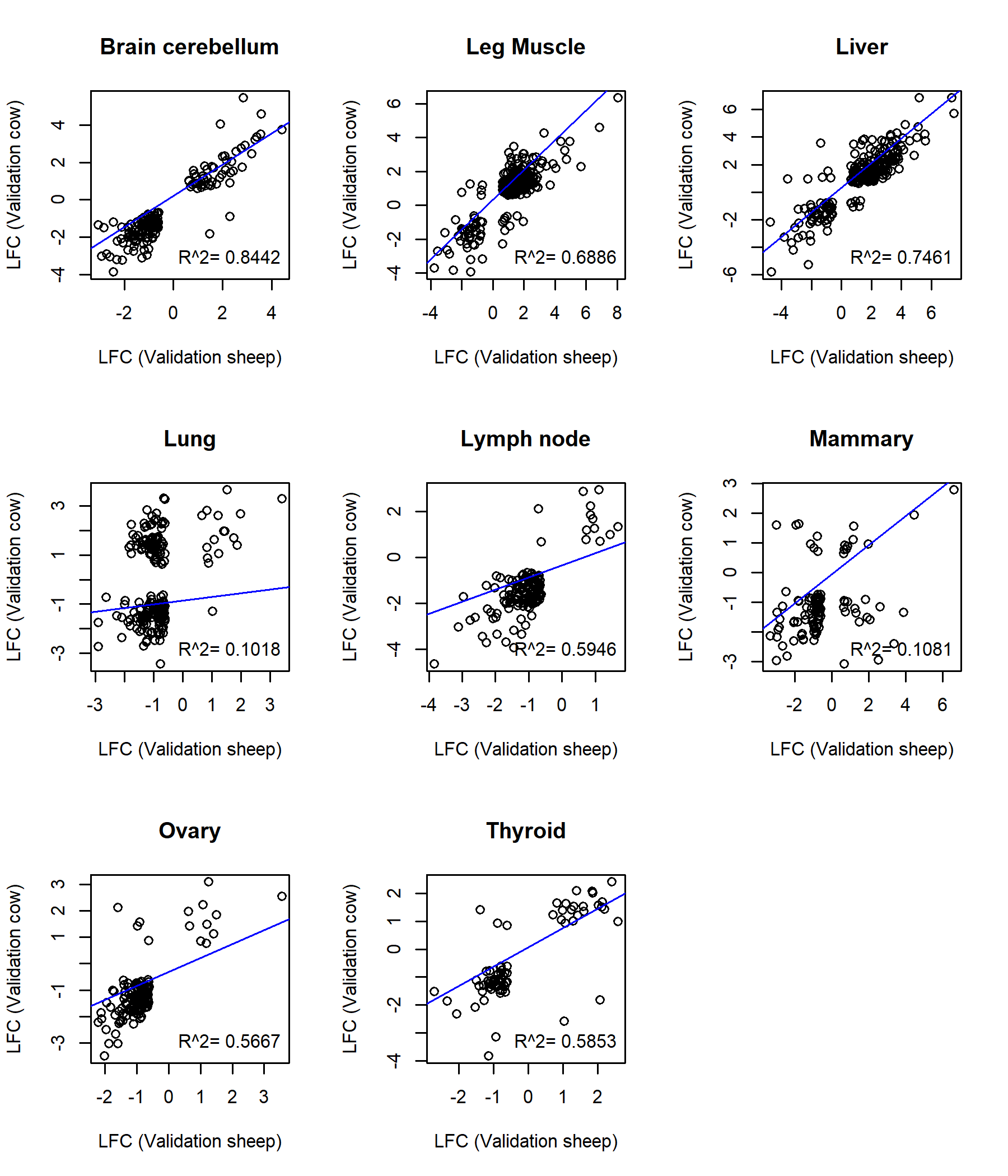

Supplement: Supplementary file 20 — Additional file 20: Figure S10. Scatter plot of log fold changes of the Validation Cow against the log-fold changes of the Validation Sheep for mitochondrial protein gene expression. [file 12864_2020_7018_MOESM20_ESM.tiff]

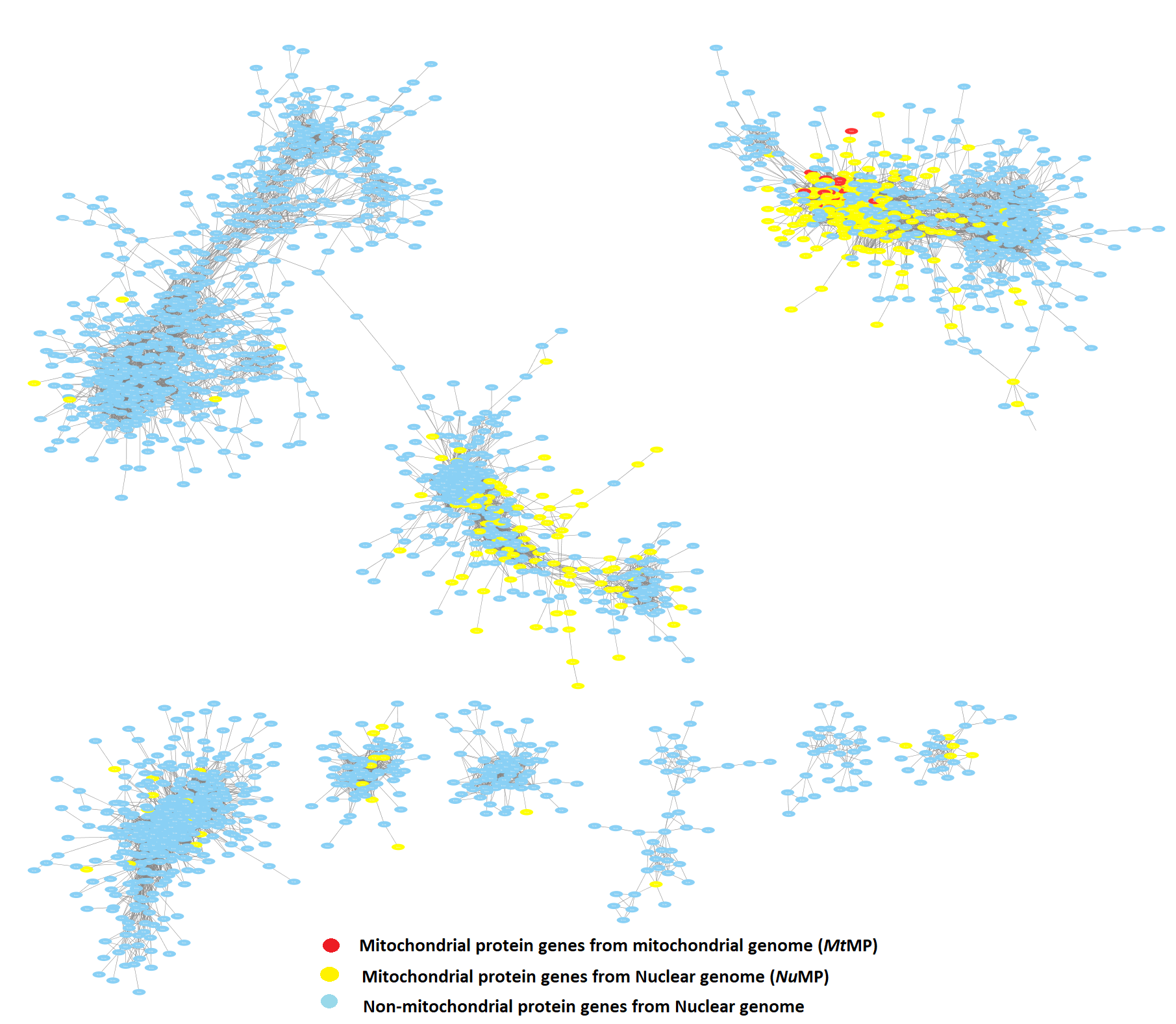

Supplement: Supplementary file 21 — Additional file 21: Figure S11. Gene co-expression network constructed based similarity matrix computed using Person Correlation Co-efficient of gene expression at r > |0.95| across tissues of the Validation Cow. [file 12864_2020_7018_MOESM21_ESM.tiff]

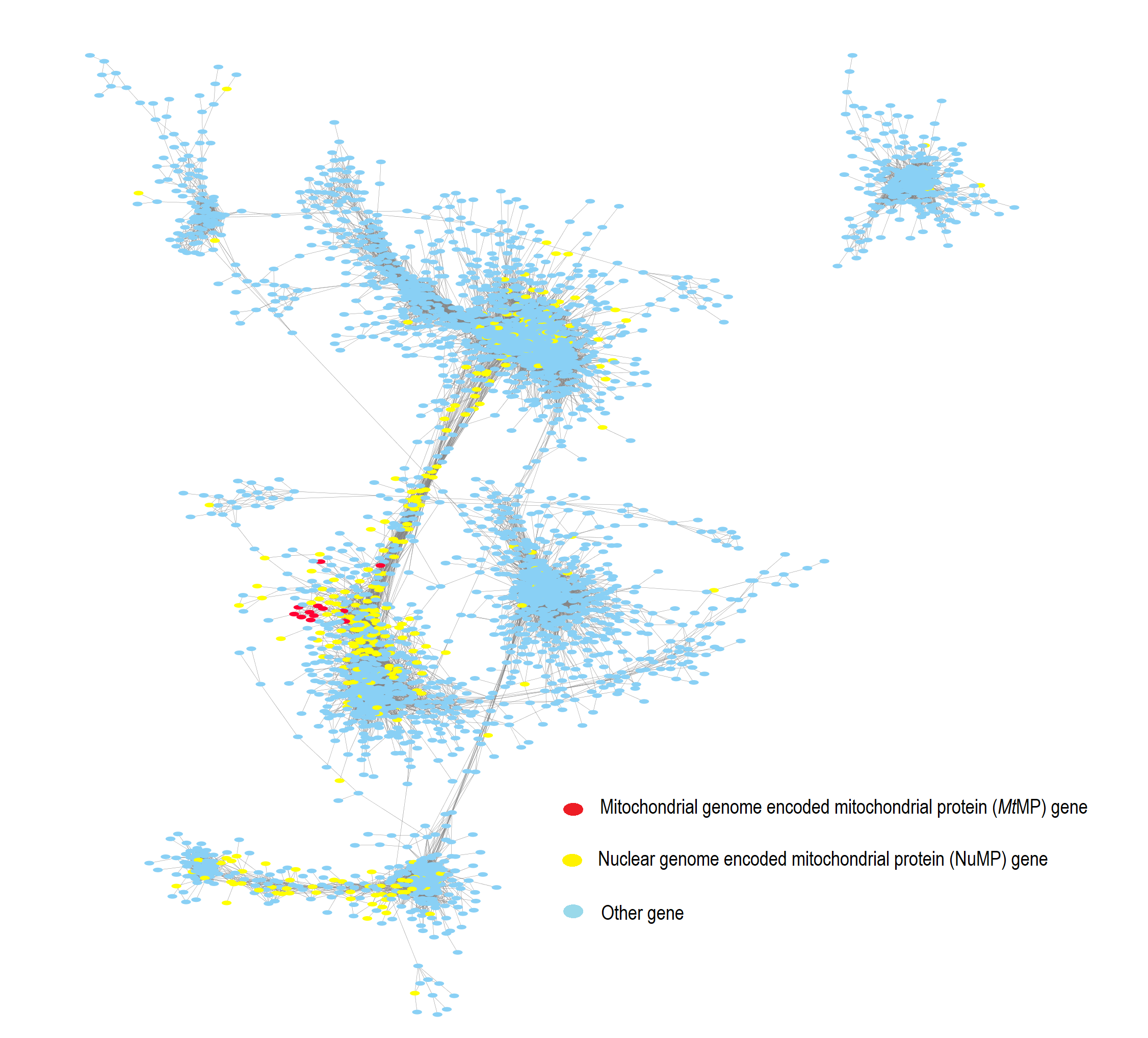

Supplement: Supplementary file 22 — Additional file 22: Figure S12. Gene co-expression network constructed based similarity matrix computed using Person correlation coefficient of gene expression at r > |0.95| across tissues of the Validation Sheep (three Texel x blackface adult female sheep AF1, AF2 and AF3). [file 12864_2020_7018_MOESM22_ESM.tiff]
