# Supplementary figures and images for: Correction to: Expression of mitochondrial protein genes encoded by nuclear and mitochondrial genomes correlate with energy metabolism in dairy cattle
Source: BMC Genomics. 2022 Apr 20;23:315. doi: 10.1186/s12864-022-08404-z (PMC9022241; doi:10.1186/s12864-022-08404-z)

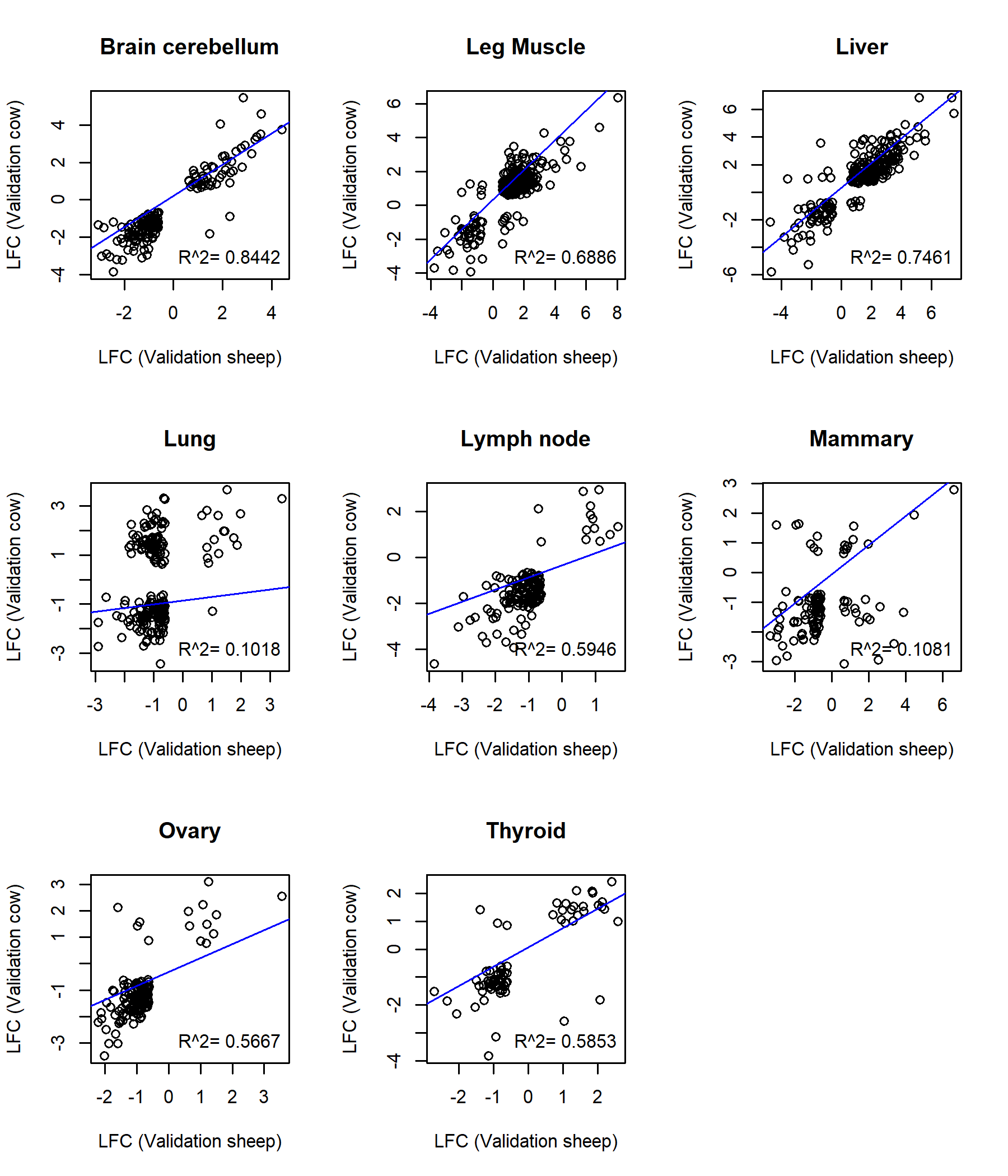

Supplement: Supplementary file 11 — Additional file 20: Figure S10. Scatter plot of log fold changes of the Validation Cow against the log-fold changes of the Validation Sheep for mitochondrial protein gene expression. [file 12864_2022_8404_MOESM11_ESM.tiff]
